# Generation of functional human oligodendrocytes from dermal fibroblasts by direct lineage conversion

**DOI:** 10.1242/dev.199723

**Published:** 2022-06-24

**Authors:** Koji Tanabe, Hiroko Nobuta, Nan Yang, Cheen Euong Ang, Philip Huie, Sacha Jordan, Michael C. Oldham, David H. Rowitch, Marius Wernig

**Affiliations:** 1 I Peace, Inc, Palo Alto, CA 94303, USA; 2 Institute for Stem Cell Biology and Regenerative Medicine, Stanford University School of Medicine, Stanford, CA 94305, USA; 3 Eli and Edythe Broad Center of Regeneration Medicine and Stem Cell Research, University of California San Francisco, San Francisco, CA 94143, USA; 4 Department of Bioengineering, Stanford University School of Medicine, Stanford, CA 94305, USA; 5 Department of Pathology, Stanford University School of Medicine, Stanford, CA 94305, USA; 6 Department of Surgical Pathology, Stanford Health Care, Palo Alto, CA 94305, USA; 7 Center for Advanced Biotechnology and Medicine, Rutgers University, Piscataway, NJ 08854, USA; 8 Department of Neurological Surgery, University of California San Francisco, San Francisco, CA 94143, USA; 9 Departments of Pediatrics and Neurosurgery, University of California San Francisco, San Francisco, CA 94143, USA; 10 Department of Paediatrics and Wellcome Trust-Medical Research Council Cambridge Stem Cell Institute, University of Cambridge, Hills Road, Cambridge CB2 0QQ, UK

**Keywords:** Direct lineage conversion, Oligodendrocyte, Leukodystrophy, Myelination, Fibroblasts, Human

## Abstract

Oligodendrocytes, the myelinating cells of the central nervous system, possess great potential for disease modeling and cell transplantation-based therapies for leukodystrophies. However, caveats to oligodendrocyte differentiation protocols ( Ehrlich et al., 2017; Wang et al., 2013; Douvaras and Fossati, 2015) from human embryonic stem and induced pluripotent stem cells (iPSCs), which include slow and inefficient differentiation, and tumorigenic potential of contaminating undifferentiated pluripotent cells, are major bottlenecks towards their translational utility. Here, we report the rapid generation of human oligodendrocytes by direct lineage conversion of human dermal fibroblasts (HDFs). We show that the combination of the four transcription factors *OLIG2*, *SOX10*, *ASCL1* and *NKX2.2* is sufficient to convert HDFs to induced oligodendrocyte precursor cells (iOPCs). iOPCs resemble human primary and iPSC-derived OPCs based on morphology and transcriptomic analysis. Importantly, iOPCs can differentiate into mature myelinating oligodendrocytes *in vitro* and *in vivo.* Finally, iOPCs derived from patients with Pelizaeus Merzbacher disease, a hypomyelinating leukodystrophy caused by mutations in the proteolipid protein 1 (*PLP1*) gene, showed increased cell death compared with iOPCs from healthy donors. Thus, human iOPCs generated by direct lineage conversion represent an attractive new source for human cell-based disease models and potentially myelinating cell grafts.

## Abstract (precis)

**Summary:** This paper shows the generation of myelinating human oligodendrocytes from dermal fibroblasts using a direct lineage conversion technique, which could be of high interest for regenerative medicine applications.

## INTRODUCTION

Oligodendrocytes, the myelinating glia cells of the central nervous system, are required for the proper saltatory conductance of neuronal action potentials (Nave and Werner, 2014). Diseases affecting myelin such as Pelizaeus Merzbacher disease (PMD) and other leukodystrophies, as well as the autoimmune disorder multiple sclerosis (Buzzard et al., 2017), result in disruption of myelin, leading to neurological dysfunction (Woodward, 2008). The finding that myelin can be restored by endogenous or transplanted oligodendrocyte precursor cells (OPCs) (Nobuta et al., 2019; Gupta et al., 2012; Uchida et al., 2012) raises the possibility of their use in cell-based remyelination therapies. However, generating autologous human OPCs in sufficient numbers for transplantation is a major hurdle for the development of myelin-restoring cell-based therapies. Embryonic stem cells (ESCs) and induced pluripotent stem cells (iPSCs) can be differentiated into functional OPCs (Ehrlich et al., 2017; Wang et al., 2013; Douvaras and Fossati, 2015), but current protocols are inefficient, slow and heterogeneous. Previously, we and others have demonstrated that OPCs can also be generated by direct conversion of fibroblasts from rodents (Yang et al., 2013; Najm et al., 2013). These induced OPCs (iOPCs) showed cell biological and molecular features of primary OPCs and gave rise to myelinating oligodendrocytes upon transplantation into the myelin-deficient Shiverer mouse brain. Based on these promising studies in rodents, we here set out to develop a method to generate iOPCs from human dermal fibroblasts.

## RESULTS

### Rodent iOPC reprogramming protocol does not support human fibroblast reprogramming

Assuming that rodent and human oligodendrocyte biology are fundamentally similar, we hypothesized that the same combination of transcription factors that successfully convert mouse and rat fibroblasts to iOPCs would also function in human cells. We therefore expressed *Olig2*, *Sox10* and *Zfp536* or *Olig2*, *Sox10* and *Nkx6.1* (also known as *Nkx6-1*) in human neonatal fibroblasts using lentiviral gene delivery and evaluated whether oligodendrocyte features would be induced. In contrary to our hypothesis, we could not detect any morphological features of OPCs in either condition tested (Fig. S1A), nor did we observe a robust induction of oligodendrocyte markers.

### A computational approach identifies novel oligodendrocyte-specific transcription factors

We therefore hypothesized that induction of oligodendrocyte identity in human cells may require different or additional transcription factors. To identify candidate factors, we performed gene co-expression analysis of laser-microdissected bulk samples from mid-gestation human neocortex (Miller et al., 2014). Previous work has shown that this approach can reveal gene co-expression signatures of distinct cell types driven by variation in cellular composition among bulk samples (Oldham et al., 2008; Kelley et al., 2018). This analysis revealed a group of co-expressed genes that contained well-known OPC and oligodendrocyte markers (Fig. S2A,B), as well as six candidate transcription factors (*SP5*, *NR0B1*, *TBX1*, *HMGA2*, *SIX4*, *DBX2*) that may be involved in human OPC development (Fig. S2C). However, when tested in combination with the established rodent reprogramming factors, none of these factors enhanced human iOPC generation (Fig. S2D).

### Successful generation of iOPCs from human fibroblasts

**Fig. 1.**
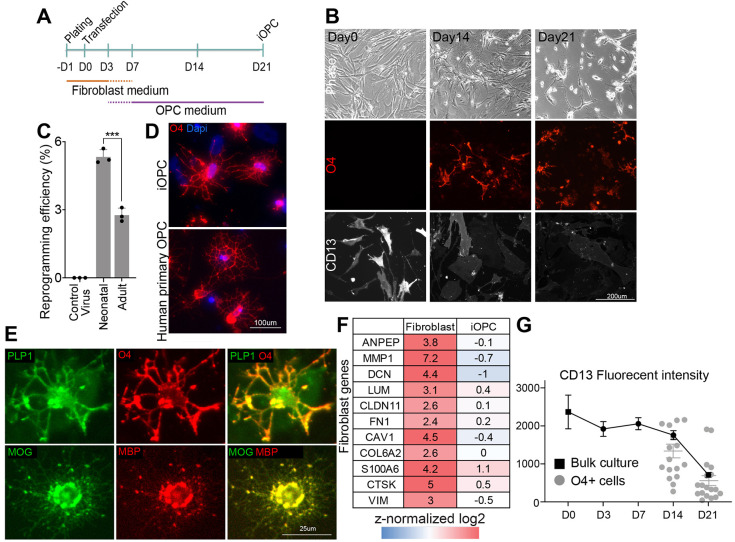
**Successful generation of iOPCs from human fibroblasts.** (A) Schematic of the optimized iOPC lineage conversion protocol. Upon retroviral infection with conversion TFs on day 0, iOPCs can be obtained on day 21. (B) Top row: morphological changes in OSAN2/6 five-factor infected HDFs over time. Middle row: O4+ cells appear at around day 14. Bottom row: the fibroblast marker CD13 decreases over time. (C) HDFs from both adult and neonatal sources are capable of generating iOPC, with higher efficiency in neonatal sources [*N*=3 foreskin fibroblast cultures from three different neonates, *N*=3 different dermal fibroblasts from three different adults. Error bars indicate mean±s.d. ***P<0.001 (unpaired two-tailed *t*-test)]. (D) iOPCs at day 21 morphologically resemble human primary OPCs. (E) O4+ iOPCs express the additional oligodendrocyte marker PLP1 (top row) and, following a 30-day differentiation period in media lacking growth factors, isolated iOPCs express the terminal differentiation markers MOG and MBP. (F) Fibroblast marker genes curated from RNA-seq analysis are downregulated in iOPCs at day 21. (G) Quantification of CD13 fluorescence intensity obtained by a CCD camera at equal settings shows a drop between day 14 and 21 in the bulk culture after infection (black line graph, bulk culture: *N*=6, error bars indicate mean±s.e.m.). Within reprogramming O4+ cells, the CD13 fluorescence intensity is even lower (gray dot plot: error bars indicate mean±s.e.m.).

We next explored different gene delivery systems. Speculating that different viruses may possess altered expression dynamics, we tested Moloney-based retroviral gene delivery. Retroviruses were also first used to generate human iPSCs with greater efficiency than lentiviruses (Takahashi et al., 2007). A direct comparison between lentivirus and retrovirus infections in human dermal fibroblasts (HDFs) showed that, although similar infection efficiency can be achieved, a significantly higher protein expression was obtained from retrovirus infection (Fig. S1B-D). Attempts to infect with increased amounts of lentivirus caused significant amount of cell death, excluding further optimization. We settled down to the five transcription factors *OLIG2*, *SOX10*, *ASCL1*, *NKX2.2* (*NKX2-2*) and *NKX6.1* (referred as OSAN2/6) based on appearance of O4+ cells as well as morphological characteristics resembling human primary OPCs during various combinatorial transcription factor (TF) applications, and cloned them into retroviral vectors. Upon infection, the fibroblasts began to change morphology within 7 days and expressed the oligodendrocyte marker O4 within 14 days (Fig. 1A,B; Fig. S1E). Owing to concurrent proliferation and cell death, as well as lack of colony formation, it was difficult to determine the scientifically accurate reprogramming efficiency (as determined by how many fibroblasts of the starting cell population will be converted into oligodendrocytes). However, determining the ratio of reprogrammed cells at a given time point relative to the number of starting cells provides practical information that is directly linked to the true reprogramming efficiency and can be used to compare different reprogramming conditions. After optimization of the culture conditions in viral titers and initial cell seeding density (Fig. S1F), the number of O4+ cells divided by the number of cells initially seeded ranged from 2.8 to 5.8% depending on the HDF line at day 21 after infection with the five transcription factors (Fig. 1C). Under these optimized conditions we consistently observed cells with oligodendrocyte-like ramified morphology resembling human primary oligodendrocytes (Fig. 1D) that expressed the oligodendrocyte marker O4 as well as PLP1 (Fig. 1E).

After purification and long-term culture without growth factors, the cells expressed terminally differentiated oligodendrocyte markers MOG and MBP (Fig. 1E). The appearance of O4 at day 21 was associated with decreases in the expression of HDF marker genes (Fig. 1F), and cells that expressed O4 typically contained lower HDF marker *ANPEP* (CD13) fluorescent intensity compared with that of the bulk average culture (Fig. 1G), suggesting an inverse correlation between fibroblast and oligodendrocyte identities. Thus, it is possible to convert human fibroblasts to cells closely resembling oligodendrocytes and their precursors.

We next sought to explore whether the induction of OPC-like cells from human fibroblasts would be restricted to this one selected fibroblast cell line or whether it can be achieved in fibroblasts derived from multiple independent donors. Indeed, we were able to generate OPC-like cells from foreskin fibroblasts from three human neonates and three adult human subjects (ages 36-67), with higher efficiency in neonatal fibroblasts presumably due to presence of relatively higher proliferative cells (Fig. 1C).

### *OLIG2*, *SOX10*, *ASCL1* and *NKX2.2* is the most efficient transcription factor combination to generate human iOPCs

**Fig. 2.**
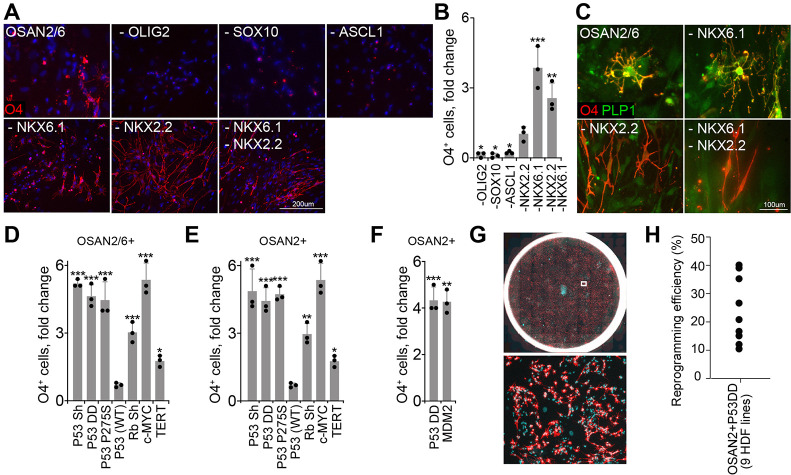
**Optimization of the reprogramming factor combination and tumor suppressor gene involvement.** (A) Representative O4 stainings after systematic elimination of one factor at a time. (B) Elimination of *OLIG2*, *SOX10* or *ASCL1* from the five-factor pool (OSAN2/6) significantly reduced the direct conversion efficiency (fold change from OSAN2/6 five-factor pool). Elimination of *NKX2.2*, *NKX6.1* or both did not reduce the efficiency (*N*=3, error bars indicate mean±s.d., one-way ANOVA, *P<0.05, **P<0.01, ***P<0.001). (C) Elimination of *NKX2.2* but not *NKX6.1* produced O4+ cells resembling fibroblasts that lack PLP1 expression. (D,E) When the P53 pathway was inhibited by short hairpin against P53 (P53 Sh) or two unique dominant-negative versions of P53 (P53 DD and P53 P275S), the fold change from OSAN2/6 (D) and OSAN2 (E) significantly increased. Upstream suppressor of P53 (Rb and c-MYC) and wild-type P53 confirmed P53 as suppressor of iOPC reprogramming. TERT showed only a modest, but significant, effect (*N*=3, error bars indicate mean±s.d., one-way ANOVA, *P<0.05, **P<0.01, ***P<0.001). (F) Overexpression of a direct negative regulator of P53, MDM2, mimicked the effect of P53DD (*N*=3, error bars indicate mean±s.d., one-way ANOVA, **P<0.01, ***P<0.001). (G) Top: representative image of O4+ iOPC induced with OSAN2-DD in 12-well plate culture format at day 21. Bottom: high magnification image of the boxed area from the top image. Red, O4; cyan, DAPI. (H) Variation of the reprogramming efficiency of nine different human dermal fibroblast lines.

In order to identify the essential minimum factors necessary for direct conversion from HDF to iOPC, we systematically eliminated one factor at a time from the five-factor pool (OSAN2/6). When *OLIG2*, *SOX10* or *ASCL1* were individually eliminated, the number of O4+ cells decreased significantly (Fig. 2A,B), indicating these three factors were required for the reprogramming process. In contrast, elimination of *NKX6.1* slightly increased the number of O4+ cells (Fig. 2A,B). Subtraction of *NKX2.2* did not change the number of O4+ cells but abolished the oligodendrocyte-like ramification in morphology, such that cells resembled the original HDFs (Fig. 2A,B). Moreover, O4+ cells without *NKX2.2* failed to express additional oligodendrocyte marker PLP1 (Fig. 2C). Elimination of both *NKX2.2* and *NKX6.1* showed similar results to the omission of *NKX2.2*. These results suggest that *NKX6.1* and *NKX2.2* cannot compensate for each other and that *NKX2.2* is important to induce faithful oligodendrocyte identity, whereas *NKX6.1* is dispensable. Taken together, these findings indicate that the four factors *OLIG2*, *SOX10*, *ASCL1*, and *NKX2.2* (OSAN2) make up the optimal transcription factor combination to convert human fibroblasts to oligodendrocytes. To improve the conversion efficiency, we explored the effects of changing the relative amounts of individual transcription factors during reprogramming. Remarkably, when the amount of *SOX10* virus was doubled, the relative number of oligodendrocytes significantly increased, whereas increasing any other factors had no effect (Fig. S3A,B). Thus, the relative expression levels of the four reprogramming factors matters for iOPC reprogramming, and high levels of SOX10 may be the most essential.

### *P53* is a crucial inhibitor of iOPC reprogramming

Retroviral gene delivery allowed the robust generation of oligodendrocytes from fibroblasts, but a higher conversion efficiency would greatly benefit translational applications. We reasoned that known reprogramming suppressors in HDF-to-iPSC conversion, such as *P53* (*TP53*) (Hong et al., 2009), might also be active during direct conversion from HDF to OPC. Indeed, suppression of *P53* during direct conversion with a retrovirus encoding a small hairpin *P53* impressively increased the conversion efficiency by about 5-fold (Fig. 2D,E; Fig. S4A,B). Suppression of *P53* was associated with increased HDF proliferation only at day 3 of reprogramming and became insignificant at later phases of reprogramming (day 14-21). In contrast, it significantly reduced HDF cell death from day 3 to day 14 of reprogramming, suggesting its stronger role in cell death inhibition (Fig. S4C). We confirmed the benefit of functional *P53* suppression with two additional reagents, a carboxy-terminal dominant negative fragment *P53*DD (Bowman et al., 1996) and the dominant negative *P53* mutant Pro275Ser (De Vries et al., 2002) (Fig. 2D,E; Fig. S4A). Knockdown of the cell cycle and chromatin regulator *Rb* (*RB1*) also improved the conversion efficiency but not to the same extent as loss of *P53* function (Fig. 2D,E), a similar observation as in iPSC reprogramming (Kareta et al., 2015). Finally, co-expression of the oncogene *c-MYC* (*MYC*) also improved the reprogramming efficiency to a similar degree as *P53* manipulation, and overexpression of *TERT* had a modest, but significant, effect (Fig. 2D,E). As expected, the loss-of-function effects of *P53* could be well recapitulated with overexpression of *MDM2*, a direct negative *P53* regulator (Fig. 2F). However, gain of *P53* function by overexpression did not result in a significant change (Fig. 2D,E; Fig. S4A). The effects of the manipulation of cell cycle regulators were similar between the OSAN2/6 or OSAN2 factor combination reprogramming (Fig. 2D,E).

### Improved iOPC reprogramming is reproducible among different donors

Again, we next confirmed that this optimized reprogramming approach is applicable to fibroblasts from multiple donors. We tested fibroblasts from nine donors and all could be successfully reprogrammed using OSAN2 and *P53*DD, with a reprogramming efficiency ranging from 11.5 to 40.0%, as calculated by the number of O4+ cells divided by the number of cells initially seeded (2.5×10^3^ per cm^2^) at day 21 (Fig. 2G,H). These results establish that the combination of lineage-specific transcription factors with manipulation of cell cycle regulators such as *P53* can robustly convert fibroblasts from many individuals to iOPCs with relatively high efficiencies.

### Human iOPCs share a similar expression profile with primary OPCs

**Fig. 3.**
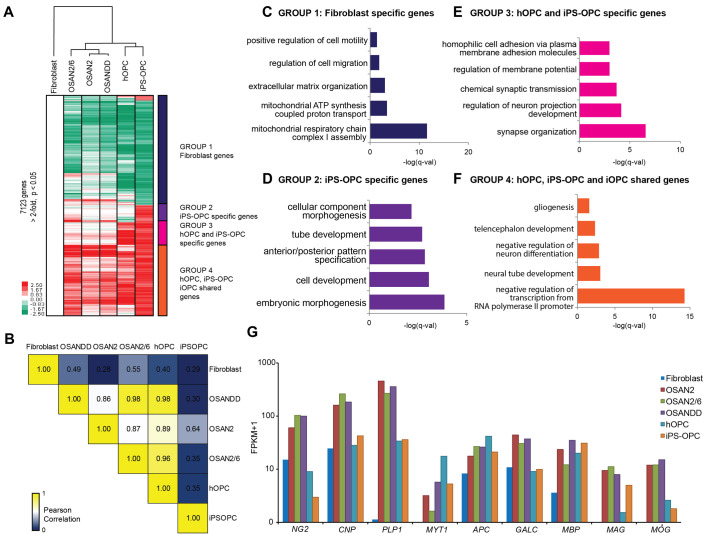
**Genome-wide remodeling of the iOPC transcriptome.** (A) Hierarchical clustering of the 7123 genes that are at least 2-fold different (*P*<0.05) between human fibroblasts, five-factor iOPC (OSAN2/6), four-factor iOPC (OSAN2), four-factor iOPC with P53 inhibition (OSAN-DD), human forebrain purified primary OPC (hOPC) and human iPSC-derived OPCs (iPSC-OPC), normalized to fibroblasts. The colored bar to the right of the heatmap separates the genes into four different groups (fibroblast specific, iPSC-OPC specific, hOPC/iPSC-OPC specific and iOPC/hOPC/iPSC-OPC specific genes). Note the three different iOPC conditions cluster closer to the hOPC and iPSC-OPC than to fibroblasts. (B) Pairwise Pearson correlation among the different iOPC samples. (C-F) GO terms for each of the groups described in A. (G) Selected OPC genes from RNA-seq confirm upregulation in the iOPC samples compared with HDFs.

To obtain a global transcriptional characterization of these iOPCs, we next compared the global gene expression of iOPCs to HDFs, human forebrain-derived primary OPC (hOPC) and human iPSC-derived oligodendrocytes (iPSC-OPC) (Fig. 3A,F,G). The heatmap depicting differentially expressed genes between hOPC and HDFs (7123 genes, >2-fold, *P*-value<0.05) showed global reprogramming of the HDF transcriptome towards that of a primary human oligodendrocyte lineage (Fig. 3A). Indeed, supervised hierarchical clustering analysis showed that transcriptional profiles of iOPCs (OSAN2/6, OSAN2 and OSAN2-DD) are much more similar to primary or iPSC-derived oligodendrocytes than HDFs (Fig. 3A). Pearson's correlation analysis of the expression values of all differentially expressed genes showed that the transcriptional profile of iOPCs is more similar to that of primary OPCs (*r*^2^=0.89-0.98) than to that of iPSC-OPCs (*r*^2^=0.30-0.64) or fibroblasts (*r*^2^=0.28-0.55) (Fig. 3B). In agreement with these findings, genes upregulated in iOPCs compared with HDFs were enriched for Gene Ontology (GO) terms associated with oligodendrocyte development such as gliogenesis and negative regulation of neuron differentiation (Fig. 3F). Conversely, genes downregulated in iOPCs compared with HDFs included GO terms associated with HDF functions such as extracellular matrix, positive regulation of cell motility and regulation of cell migration (Fig. 3C). Of note, there were genes specific to iPSC-OPC, enriched for GO terms such as anterior-posterior patterning genes (e.g. HOX genes) (Fig. 3D), possibly reflecting the posteriorizing effect of the iPSC-OPC differentiation protocol which involves retinoic acid-containing media (Douvaras and Fossati, 2015). Those posterior HOX genes were not expressed in either hOPC or iOPC samples. On the other hand, genes specific to hOPC and iPSC-OPC were enriched for neuronal GO terms, indicating gene expression changes potentially caused by interaction with neurons (which are absent in iOPC cultures) or even a possible neuronal contamination in these samples (Fig. 3E). Manual inspection of oligodendrocyte-specific genes *NG2* (*CSPG4*), *CNP*, *PLP1*, *MYT1*, *APC*, *GALC*, *MBP*, *MAG* and *MOG* confirmed increased expression in iOPC and iPSC-derived oligodendrocytes compared with HDFs or HDFs cultured in control iOPC condition (Fig. 3G; Fig. S5A,B). Expression changes of these genes and genes used in direct conversion were confirmed by qPCR (Fig. S5A,B).

### Human iOPCs differentiate into myelinating oligodendrocytes *in vivo*

**Fig. 4.**
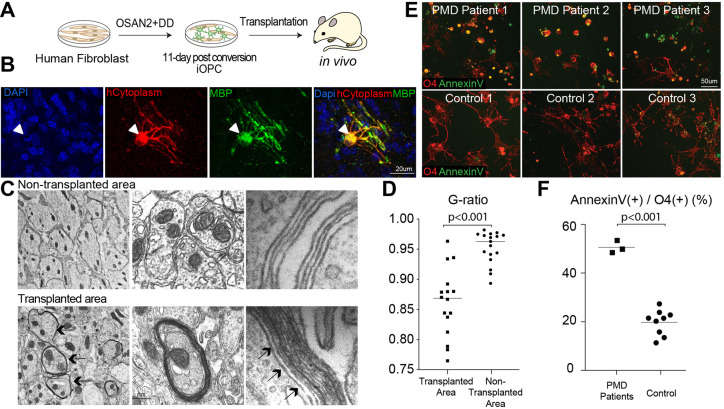
**Engraftment and myelination *in vivo* and disease modeling capability of iOPCs.** (A) To test the functional efficacy of iOPC, cells were transplanted into the cerebellum of postnatal immune-deficient Shiverer mice (*N*=3). (B) MBP (green) and the human-specific antibody SC121 (hCytoplasm, red) detected iOPC-derived cells with a typical morphology of mature oligodendrocytes 12 weeks after transplantation. (C) Electron microscopy of the transplantation area. Top row: only thinly and loosely myelinated axons were found in Shiverer mouse. Bottom row: electron microscopy of the area transplanted with iOPCs revealed multi-layer, compact myelin (arrows). (D) G-ratio quantification of axons in transplanted and non-transplanted areas (*N*=16-17, unpaired two-tailed *t*-test, *P*<0.001). (E) Pelizaeus Merzbacher disease patient-derived fibroblasts and healthy controls were directly converted to iOPCs. At day 21, patient iOPCs showed morphological abnormalities and increased cell death detected by annexin V. (F) Quantification of annexin V staining in iOPCs (*N*=3 for patient, *N*=9 for control, unpaired two-tailed *t*-test, *P*<0.001).

To test the myelinating capability of iOPC, we conducted an *in vivo* transplantation study in the Shiverer mouse, which lacks compact myelin throughout the central nervous system due to deletion of the *Mbp* gene (Roach et al., 1983). Reprogramming cultures were harvested on day 11 following infection with the OSAN2-DD factor combination and stereotaxically transplanted into the cerebellum of Shiverer mouse on postnatal day 1 (Fig. 4A). At 12-13 weeks post transplantation, we observed several areas with engrafted cells of human origin that expressed the mature oligodendrocyte marker MBP (Fig. 4B). Electron microscopic analysis revealed axons with multi-layer compact myelin in the region transplanted with iOPCs (G-ratio 0.860±0.0563); these were not present in untransplanted areas (G-ratio 0.9527±0.00268, *N*=16-17, unpaired two-tailed *t*-test, *P*<0.001) (Fig. 4C). We observed no incidence of tumor formation in transplanted hosts (*N*=8).

### iOPCs can be used to model genetic myelin diseases

Taking advantage of the rapid direct conversion timeline (<21 days) of the iOPC protocol, we tested the feasibility of its use in white matter disease modeling. PMD is a hypomyelinating leukodystrophy caused by mutations in the *PLP1* gene, encoding an abundant myelin protein (Woodward, 2008). Currently, patient-specific modeling of PMD relies on conventional differentiation of iPSCs to OPCs (Nobuta et al., 2019; Numasawa-Kuroiwa et al., 2014) or myelin containing cerebral spheroids (Madhavan et al., 2018), taking 55 or more than 210 days, respectively. We obtained fibroblast biopsies from three patients with PMD harboring unique mutations in *PLP1*. Their defective myelination and cell death have been described as part of a clinical study (Gupta et al., 2012) and an iPSC-based disease modeling study (Nobuta et al., 2019). The patient HDFs converted to iOPC following the OSAN2+P53DD method showed a significant increase of the apoptosis marker annexin V compared with nine control cultures at day 21 (Fig. 4D,E). These findings are very similar to previous observations made in iPSC-OPCs (Nobuta et al., 2019). Thus, iOPCs directly reprogrammed from dermal fibroblasts retain patients’ disease phenotypes and can serve as a rapid disease modeling system completed in 21 days.

## DISCUSSION

Here, we have developed an optimized retroviral transfection platform and showed that the four-TF pool (OSAN2) directly converts human HDFs to oligodendrocyte lineage cells, which resemble their primary counterparts transcriptionally and functionally. Upon realizing that the TF combinations reported for mouse and rat fibroblasts were insufficient to convert human fibroblasts, we sought to identify a comprehensive list of human OPC genes. We employed a computational approach that identifies cell type-specific genes by analyzing gene co-expression relationships in intact tissue samples (Kelley et al., 2018). This approach identified a list of known and previously unreported OPC-specific genes which were not recognized as such before, including a number of TFs. However, none of these TFs improved induction of OPCs, suggesting candidate genes based on the specificity to OPCs (whether or not highly expressed) and overexpressing such genes does not enhance reprogramming. Nevertheless, the newly identified genes may have important functions for oligodendrocyte biology that would be worth studying in the future.

Remarkably, the eventually successful reprogramming was achieved with a pool of well known and highly expressed oligodendrocyte genes, not all of which are specifically expressed by cells in the oligodendrocyte lineage. A notable example is *ASCL1*, which earlier during development is expressed in ventricular zone progenitor cells as they transition into basal progenitors and postmitotic neurons (Wilkinson et al., 2013). Accordingly, it is a potent inducer of neuronal identity (Guillemot, 2005). However, *ASCL1* is also expressed in transit amplifying cells derived from adult subventricular neural stem cells, proliferative OPCs, dividing glioma cells and even neuroendocrine cells of endodermal tissues such as the lung, and is crucially involved in small lung cancer formation (Paliouras et al., 2012; Battiste et al., 2007; Park et al., 2017; Altree-Tacha et al., 2017). In contrast, *in vivo* expression of *ASCL1* promotes OPC formation (Jessberger et al., 2008), providing direct evidence that this factor is instructive for oligodendrocyte lineage specification. This is in accordance with its importance for oligodendrocyte specification and differentiation especially in the spinal cord together with *NKX2.2* (Sugimori et al., 2008; Fu et al., 2002; Vue et al., 2014; Parras et al., 2007). In addition, *ASCL1* and *NKX2.2* are highly expressed genes in the OPC population along with our other selected genes, according to the RNA-seq data comparing OPC, immature and mature oligodendrocytes, neurons and astrocytes (Zhang et al., 2014). The disparate functions of *ASCL1* are much debated and its phosphorylation state has been proposed to confer functional specificity (Castro et al., 2011; Ali et al., 2014; Li et al., 2014). Compatible with this model, *ASCL1*-mediated reprogramming towards neurons occurs in basal media without growth factors, whereas the media we used here to generate iOPCs contains PDGF-A, which activates MAP kinase pathways including cyclin-dependent kinases known to phosphorylate ASCL1 (Ali et al., 2014).

Another intriguing observation of this study was that higher levels of SOX10 improve reprogramming but increasing the dosage of other reprogramming factors had little effect. This may speak to the importance of *SOX10* to pioneer the chromatin landscape for the other transcription factors to properly induce the oligodendrocyte lineage. Sox transcription factors have been reported to possess pioneer factor activity (Soufi et al., 2012, 2015) and the initial chromatin opening may be the rate-limiting step, which can be accelerated by increasing the amount of exogenous transcription factors. It may not be coincidental that a higher dose of SOX2 improves iPSC reprogramming, a phenomenon which is well documented but to this day not well understood (Papapetrou et al., 2009).

In the efforts to improve the direct conversion efficiency, we noted significant effects of P53 inhibition (Fig. 2). P53, originally observed as an inhibitor of iPSC generation through suppression of proliferation and the pluripotent gene network (Hong et al., 2009; Hanna et al., 2009), has been reported to improve also the direct lineage conversions to neurons, even though neurons are postmitotic (Xu et al., 2016; Jiang et al., 2015; Sun et al., 2014). Our results on the additional beneficial effect in iOPC reprogramming suggest that P53 may represent a more general gate keeper of lineage fidelity.

Currently, human oligodendrocyte disease modeling relies on conventional differentiation protocols from iPSCs. Compared with the time required to generate iPSCs (weeks) followed by oligodendrocyte differentiation protocol (months), the direct iOPC conversion is completed in a mere 21 days with a reasonable efficiency when our optimized protocol is applied. The iOPC system can be initiated with a small number of fibroblasts (2.5×10^3^ per cm^2^), which can be obtained from routine skin biopsies, allowing a simple scale-up to accommodate a large number of patients. Importantly, we demonstrate here in a proof-of-concept study that iOPCs can recapitulate disease phenotypes that were previously established using iPSCs (Nobuta et al., 2019). Thus, the iOPC system offers a time- and cost-effective disease modeling platform.

Oligodendrocyte disorders such as congenital leukodystrophies, multiple sclerosis and cerebral white matter injury associated with premature birth are considered candidates for cell-based transplantation therapy because of oligodendrocyte ability to migrate and regenerate the damaged myelin. Thus, generating autologous transplantable human OPCs at high quantity has clinical relevance. We showed here that, despite the relatively low reprogramming efficiency, the iOPCs transplanted *in vivo* survive and engraft the host brain, and ultimately construct compact multi-layered myelin (Fig. 4A-C). This is a proof of concept that iOPCs can be used as transplantable functional cells. Recently, a similar one-step conversion has been published using *OLIG2*, *SOX10* and *NKX6.2* as the key TF combination to generate iOPCs; however, these cells have not been evaluated *in vivo* (Chanoumidou et al., 2021). Future experiments will be needed to compare iOPCs generated with these three factors compared with the TF combination we found to be most optimal, as our data suggest that *ASCL1* greatly improves the reprogramming efficiency, and cells generated with *NKX6.2* as opposed to *NKX2.2* do express OPC markers but lack morphological features typical of OPCs. It will be of high interest for regenerative medicine applications to test as well as improve the myelinating capability of these various engineered iOPCs.

## METHODS

### Cell culture and reprogramming

HDFs from three PMD patients enrolled in a clinical study (Clinicaltrials.gov identifier NCT01005004) (Gupta et al. 2012, 2019) were obtained under approval from the University of California, San Francisco Institutional Review Board (IRB) (Study Protocol 13-10806). Control HDFs from adult and neonatal sources were obtained from Cell Applications. HDFs were cultured in Dulbecco's modified Eagle medium (DMEM; Thermo Fisher Scientific) containing 10% fetal bovine serum (Thermo Fisher Scientific) and 1% Penicillin-Streptomycin (Thermo Fisher Scientific). PLAT-GP cells (Cell Biolabs) were cultured in DMEM containing 10% fetal bovine serum, 0.1% Normocin (Thermo Fisher Scientific) and 0.5% Penicillin-Streptomycin. Mycoplasma contamination has been confirmed negative in all lines.

Except the experiment described in Fig. S1F, we used neonatal fibroblasts of various sources throughout the manuscript. We introduced retroviral vectors into PLAT-GP cells using the FuGENE 6 transfection reagent (Roche) as per the manufacturer's protocol to generate retroviral particles of each reprogramming factor separately. Next day, the medium was replaced with fresh medium and incubated overnight. The supernatant containing the virus was collected and filtrated through a 0.45 μm filter (Whatman). The supernatant of each reprogramming factor was mixed with Polybrene (4 μg/ml) (Sigma-Aldrich) and incubated under centrifugation (700 ***g***, 1 h, room temperature) for spinfection as described in a previous paper (Takahashi et al. 2007). The medium was replaced with fresh medium after spinfection. This virus production and infection protocol resulted in over 75% of HDFs infected 3 days after infection by each virus. Three days later, medium was changed to PDGF medium containing DMEM/F12, N2 (1×), B27 (1×), Penicillin-Streptomycin (1×), NEAA (1×), Insulin (25 µg/ml, Sigma-Aldrich), PDGF-AA (10 ng/ml, Peprotech), IGF (10ng/ml, Peprotech), NT3 (1 ng/ml, Peprotech), Biotin (100 ng/ml, Sigma-Aldrich) and cAMP (1 µM, Sigma-Aldrich). Half of the PDGF medium was changed every other day until day 21. For terminal differentiation of iOPCs, Glia medium containing DMEM/F12, N2 (1×), B27 (1×), Penicillin-Streptomycin (1×), NEAA (1×), Insulin (25 µg/ml, Sigma-Aldrich), T3 (40 ng/ml, Sigma-Aldrich), Ascorbic Acid (20 µg/ml, Sigma-Aldrich), Biotin (100 ng/ml, Sigma-Aldrich) and cAMP (1 µM, Sigma-Aldrich) was used. Half of the Glia medium was changed every other day until fixation at 51days. All reagents were purchased from Thermo Fisher Scientific unless otherwise stated.

### Plasmid construction

The open reading frames of the genes were amplified by PCR, sub-cloned into pENTR-D-TOPO (Thermo Fisher Scientific). The open reading frames were transferred to pMXs-gw using the Gateway LR reaction system (Thermo Fisher Scientific) following the manufacturer's protocol.

### *In vivo* cell transplantation

All data shown involving animal procedures were performed according to protocols approved by the Stanford University Institutional Animal Care and Use Committee. Initially, we transplanted purified O4+ cells at day 21 from the bulk culture, but this protocol did not yield long-term surviving cells. We therefore transplanted unpurified bulk cells at 11 days after OSAN2+P53DD infection at 50,000 cells/site in 0.5 µl volume on postnatal day 1 of immunocompromized *Shiverer;Rag2*^*−/−*^*;Il2rg*^*−/−*^ mice. The site of transplantation was cerebellar white matter, identified by coordinates (0.9 mm medial-lateral, 3.0 mm posterior, 1.8 mm ventral) from lamda. Animals were sacrificed by transcardiac perfusion with 4% paraformaldehyde (PFA) + 0.25% glutaraldehyde at 12-13 weeks post transplantation, and 50 μm sagittal brain sections were made with a vibratome (Leica Biosystems) and used for immunohistochemistry.

### Histology & image acquisition

After fixation of cells and brain tissues with 4% PFA or 4% PFA+0.25% glutaraldehyde (for electron microscopy-compatible samples), samples were permeabilized with 0.2% Triton (in case of intracellular antigen detection) and incubated with primary antibodies overnight at 4°C. Secondary antibodies with appropriate species were incubated at room temperature for 1 h. Nuclei were stained with DAPI. Images were captured with an inverted microscope equipped with a CCD camera (Leica Biosystems), with all imaging parameters being equal within experimental groups.

### Antibodies

The following primary antibodies were used for staining: O4 (mouse hybridoma; Sigma-Aldrich, O7139, 1:100); PLP1 (Abcam, 28486, 1:1000); MOG (mouse monoclonal hybridoma supernatant, from B. Barres, Stanford University, USA, 1:50); MBP (AbD Serotec, MCA409S, 1:1000); STEM121 (Takara Bio, Y40410, 1:1000); AnnexinV (Cell Signaling Technology, 6592S, staining protocol provided by manufacturer); CD13 (BD Biosciences, 557454, 1:100). Secondary antibodies of appropriate species were purchased from Jackson ImmunoResearch (115165020) and Thermo Fisher Scientific (A-21245, A-21424, A-11006, A-21422). All secondary antibodies were used at 1:1000.

### qPCR primers

For quantitative PCR, total RNA was isolated at the described time points with Trizol reagent (Thermo Fisher Scientific) or RNAeasy Mini Kit (Qiagen) following the manufacturer's protocols. Contaminating DNA was removed with TURBO DNA-free kit (Thermo Fisher Scientific). cDNA was obtained using the High-capacity cDNA reverse transcription kit (Thermo Fisher Scientific). SYBR green-based qPCR was conducted in LightCycler 480 with manufacturer's reagents (Roche) with the following primer sets: OLIG2 [untranslated region (UTR)] FW GGGGCCACAAGTTAGTTGGA, RV GAGGGTGTGGATTGACCCAG; SOX10 (UTR region) FW GAGGCCCCCTGATCCAATTC, RV GGGATGCGTCTCAAGGTCAT; NKX2.2 (UTR region) FW CTTGGGAGAGGGCTGAACTC, RV GACATTAACGCTGGGACGGT; MBP FW CGTCACAGAAGAGACCCTCCC, RV AGTCAAGGATGCCCGTGTCTC; CSPG4 FW CTTCACTCAGGCAGAGGTCTACGC, RV GAGGACAGCTGGAGCTCTAGGGT; CNPASE FW AAGGAGAAGAACCAGTGGCA, RV CAAGTCCATCTTCTCCCTGG; OLIG1 FW AAAGTGACCAGAGCGGATGT, RV GAGCGAGCACTTTCTGCCTA; PDGFRA FW CCTTGGTGGCACCCCTTAC, RV TCCGGTACCCACTCTTGATCTT; MOG FW AGAGAATCTCCACCGGACTT, RV AACCAAGGGTCCAAGAACCG; PLP1 FW TATCTCATCAATGTGATCCATGCCT, RV TCCTAGCCATTTTCCCAAACAAT.

### Electron microscopy

Animals were fixed using cardiac perfusion with 4% PFA containing 0.25% glutaldehyde. Alternate vibratome sections were kept for immunohistochemistry and electron microscopy (EM). Tissue selected for EM was post-fixed in buffered 4% glutaraldehyde for several days at 4°C. Regions of interest were determined by immunohistochemistry, micro-dissected, fixed in 2% osmium tetroxide and embedded in resin. Ultrathin sections were placed onto copper grids, stained with uranyl acetate and lead citrate, and examined with a JEOL 1400 Transmission Electron Microscope. Use of JOEL 1400 was supported by National Institutes of Health grant 1S10RR02678001.

### RNA-seq

Total RNA was harvested using Trizol and libraries were built using Truseq kit with ribosomal RNA depletion (Illumina). Libraries produced were sequenced using the NextSeq mid-output platform with 2×75 reads. Raw reads were then mapped to the human reference genome (hg19) using Tophat v1.3.0. RNA-seq for fibroblasts was carried out in triplicate and for other cells from pooled RNA from three replicates. Gene expression was then calculated using the cuffdiff suite in Cufflinks v2.1.1. Candidate genes which were changed more than 2-fold and passed the FDR corrected *P*-value of 0.05 were obtained by comparing human fibroblasts and human OPC. The heatmap was generated by performing hierarchical clustering using GeneCluster 3.0 (Eisen et al. 1998) and visualized using TreeView (Saldanha 2004). The GO analyses were performed with Panther. The pairwise Pearson correlation among the different samples was computed using the cor function in R. Curation of the marker genes that define the fibroblast cluster was based on marker genes enriched in the fibroblast cluster in the single cell RNA-seq we previously performed (Kareta et al., 2015), and the heatmap was generated by picking the fibroblasts genes, log2 transformed and *z*-normalized FPKM.

### Transcriptional analysis of OPCs in developing human neocortex

A publicly available transcriptional dataset from laser-microdissected samples from laminar zones of developing human neocortex (Miller et al. 2014) was used. Unsupervised gene co-expression analysis (Lui et al. 2014) was conducted to reveal a module of co-expressed genes that was significantly enriched with markers of human OPCs using one-sided Fisher's exact test (Sim et al. 2011) and summarized by its first principal component, or module eigengene. The top 15 genes ranked by their Pearson correlation to the module eigengene, or *k*_ME_ (Uchida et al. 2012) was then summarized with genome-wide distribution of standardized *k*_ME_ values (*z*-scores).

### iPSC-OPC differentiation and purification

A previously published protocol for directed iPSC differentiation to OPCs was used (Yang et al. 2013) with the following modifications: human ESC medium and human ESC medium without basic FGF were used in place of mTeSR and custom mTeSR, respectively. The concentration of SAG was 0.5 µM, T3 was 40 ng/ml, NT3 was 1 ng/ml. Penicillin-Streptomycin was omitted from N2 medium, HGF from PDGF medium, and HEPES from Glia medium.

On day 0, iPSCs (sources described previously Najm et al. 2013) were plated at 0.25×10^6^/well in a Matrigel-coated six-well plate with ESC medium without basic FGF, supplemented with dual SMAD inhibitors, RA and ROCK inhibitor thiazovivin (Santa Cruz Biotechnology). From day 1 to day 4, N2 medium was gradually increased by 25% each day, reaching 100% on day 4. On day 8, dual SMAD inhibitors were replaced with SAG. On day 12, cells were lifted, dissociated and seeded on Petri dishes for sphere formation. On day 20, the medium was changed to PDGF medium. On day 30, spheres were plated on poly-L-ornithine/laminin-coated dishes. On day 45, the medium was changed to Glia medium.

On day 55, bulk culture containing O4+ oligodendrocytes was dissociated with papain for 5 min. Single cell suspension was passed onto a series of culture dishes coated with *Bandeiraea simplicifolia* Lectin I (BSL; Vector Laboratories, L1100) to remove non-specific cells, IgM secondary antibody (Jackson ImmunoResearch, 115-005-020, 1:1000) to avoid non-specific binding, and O4 (Mouse hybridoma), to isolate O4+ OPCs. At the end of O4 binding, adherent cells were washed, detached with Trypsin and collected by centrifugation (200 ***g*** for 3 min) and directly homogenized in Trizol for RNA collection.

### Isolation of human primary OPCs

Fetal cortical tissue (gestational weeks 20 to 23) was collected from elective pregnancy termination specimens at San Francisco General Hospital with previous patient consent. Research protocols were approved by the Committee on Human Research (Institutional Review Board) at University of California, San Francisco. The brain tissues were dissociated with papain for 10 min. Single cell suspension was passed onto a series of culture dishes coated with BSL to remove microglia and endothelial cells, IgM secondary antibody to avoid non-specific binding and O4 to isolate O4+ OPCs. At the end of O4 binding, adherent cells were washed, detached with Trypsin and collected by centrifugation (200 ***g*** for 3 min). Cells were directly homogenized in Trizol for RNA collection.

### Isolation of iOPCs

On day 21 of the reprogramming, the bulk culture was detached with Trypsin. Single cell suspension was passed onto a series of culture dishes coated with CD13 to remove non-specific cells, IgM secondary antibody to avoid non-specific binding, and O4 to isolate O4^+^ OPCs. At the end of O4 binding, adherent cells were washed, detached with Trypsin and collected by centrifugation (200 ***g*** for 3 min) and directly homogenized in Trizol for RNA collection.

### Statistics

Each *in vitro* experiment was repeated at least three times. Two to four biological replicates were included in each experiment. *In vivo* transplantation experiments were replicated in 3-4 mice in each condition during optimization. For statistical analyses, two sided one-way ANOVA, unpaired two-tailed *t*-test with normal distribution, one-sided Fisher's exact test and Pearson correlation were used as appropriate: *P-value<0.05.

## Supplementary Material

Supplementary information

Reviewer comments
