# Supplementary material for: Generation of functional human oligodendrocytes from dermal fibroblasts by direct lineage conversion
Source: Development. 2022 Jun 24;149(20):dev199723. doi: 10.1242/dev.199723 (PMC9357374; doi:10.1242/dev.199723)
Supplement: Supplementary information [file develop-149-199723-s1.pdf]

Supplemental Figure 1

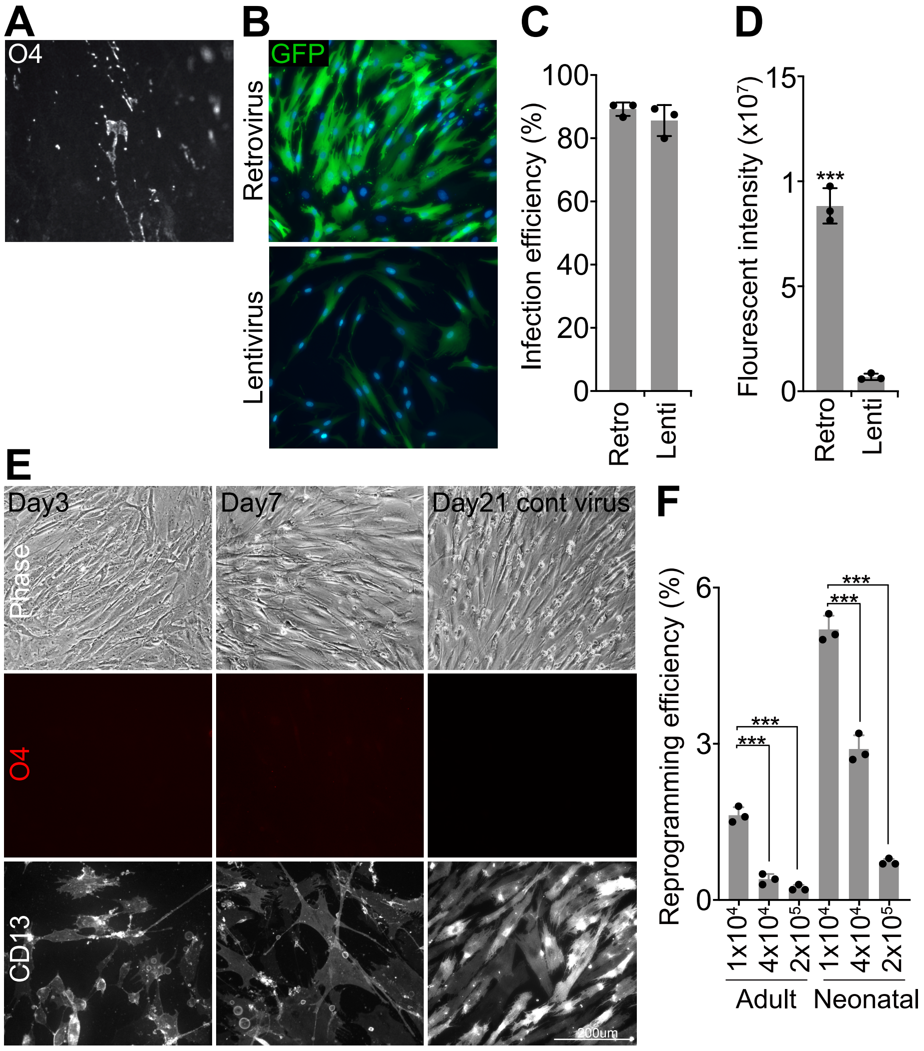

Fig. S1. Relationship between the initial seeding density and iOPC generation. (**A**) Lack of appropriate morphology in lentivirus-mediated reprogramming. (**B-D**) Comparison of infection efficiency and protein expression by retrovirus and lentivirus encoding GFP. (**E**) Top row: morphological changes in OSAN2/6 five-factor infected HDFs over time. Middle row: Lack of O4+ cells at day 3, 7, or by control virus. Bottom row: Changes in the fibroblast marker CD13 decreases over time. (**F**) Systematic assessment of initial HDF seeding density (number of cells/well in 12-well plate format) showed $1x10^4$ cells/well to be the optimal density for the highest iOPC reprogramming efficiency in both adult and neonatal HDF sources (N = 3, error bars indicate mean ± SD).

Development • Supplementary information

Suppl Figure 2

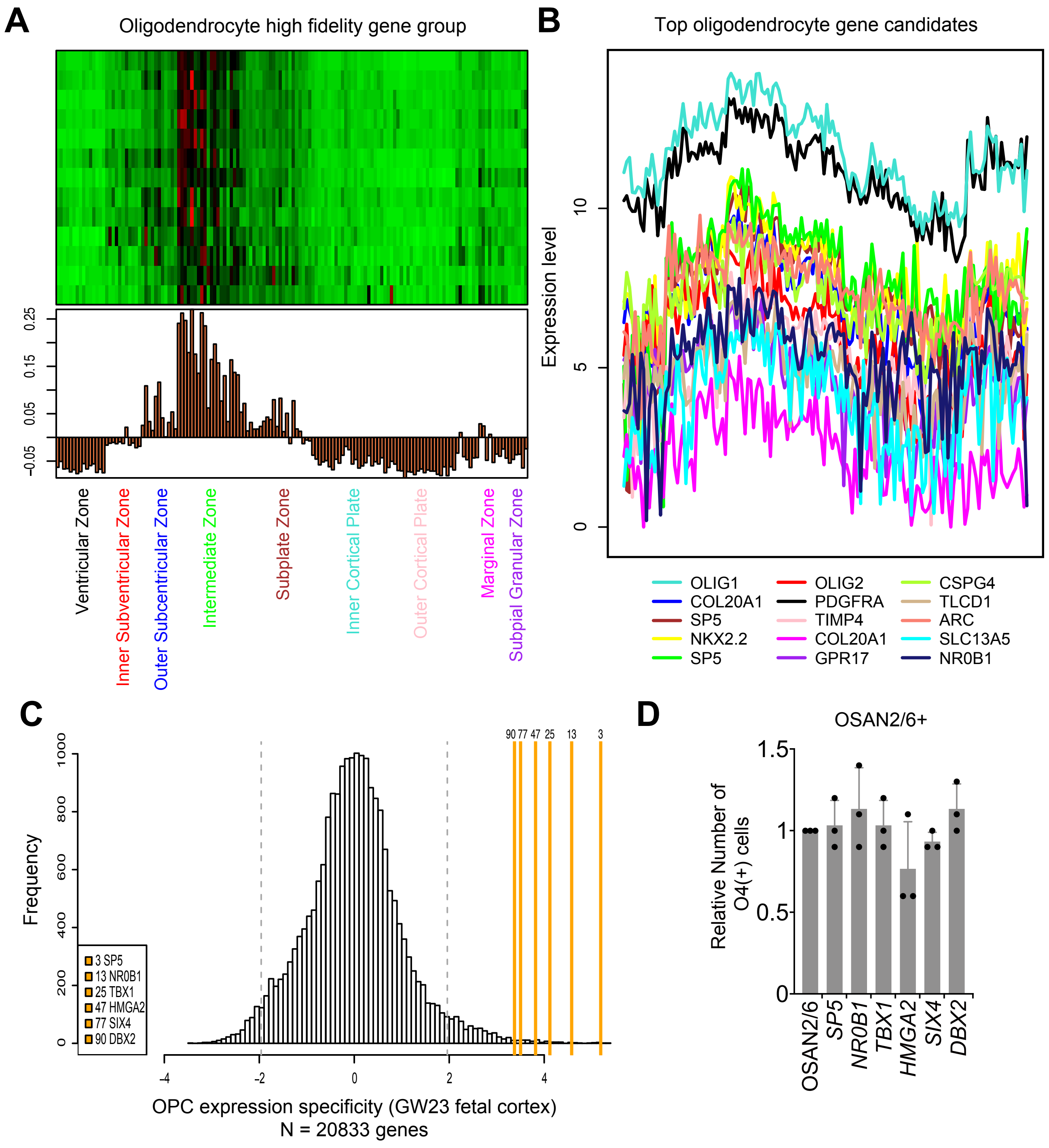

Fig. S2. A transcriptional signature of OPCs in developing human neocortex. (**A**) Top: Unsupervised gene coexpression analysis of laser-microdissected samples from laminar zones of developing human neocortex revealed a module of coexpressed genes that was significantly enriched with markers of human OPCs (p = 5.92E-11, one-sided Fisher's exact test). Bottom: the expression pattern of the module was summarized by its first principal component, or module eigengene. Note that gene expression is highest in the intermediate zone, which is presumptive white matter. (**B**) The top 15 genes ranked by their Pearson correlation to the module eigengene (a), or *k*ME. Duplicate gene symbols represent multiple microarray probes. Note *OLIG1*, *OLIG2*, *PDGFRα* , *CSPG4* (*NG2*), etc. (**C**) Genome-wide distribution of standardized *k*ME values (z-scores). Vertical lines denote locations of novel candidate TFs tested for reprogramming ability in this study. (**D**) Direct conversion efficiency for each novel TF relative to the baseline OSAN2/6 TF combination.

Development • Supplementary information

Suppl Figure 3

A

OLIG2	SOX10	ASCL1	NKX2.2	Mock
0.2	0.2	0.2	0.2	0.2
0.4	0.2	0.2	0.2	0
0.2	0.4	0.2	0.2	0
0.2	0.2	0.4	0.2	0
0.2	0.2	0.2	0.4	0

B

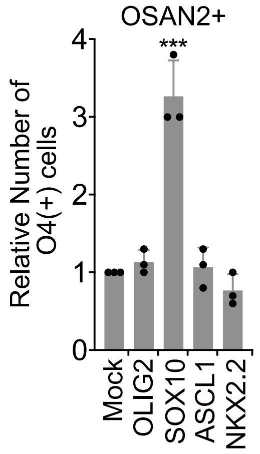

Fig. S3. Improvement of iOPC efficiency by systematic weighing of TFs. (**A**) The fraction of virus volume within 1.0 total volume was systematically doubled for each virus at a time to quantify iOPC reprogramming efficiency. (**B**) When *SOX10* fraction was doubled, there was a significant increase in iOPC efficiency (N = 3, error bars indicate mean ± SD, one way ANOVA, *** indicates p-value<0.001).

Development • Supplementary information

Suppl Figure 4

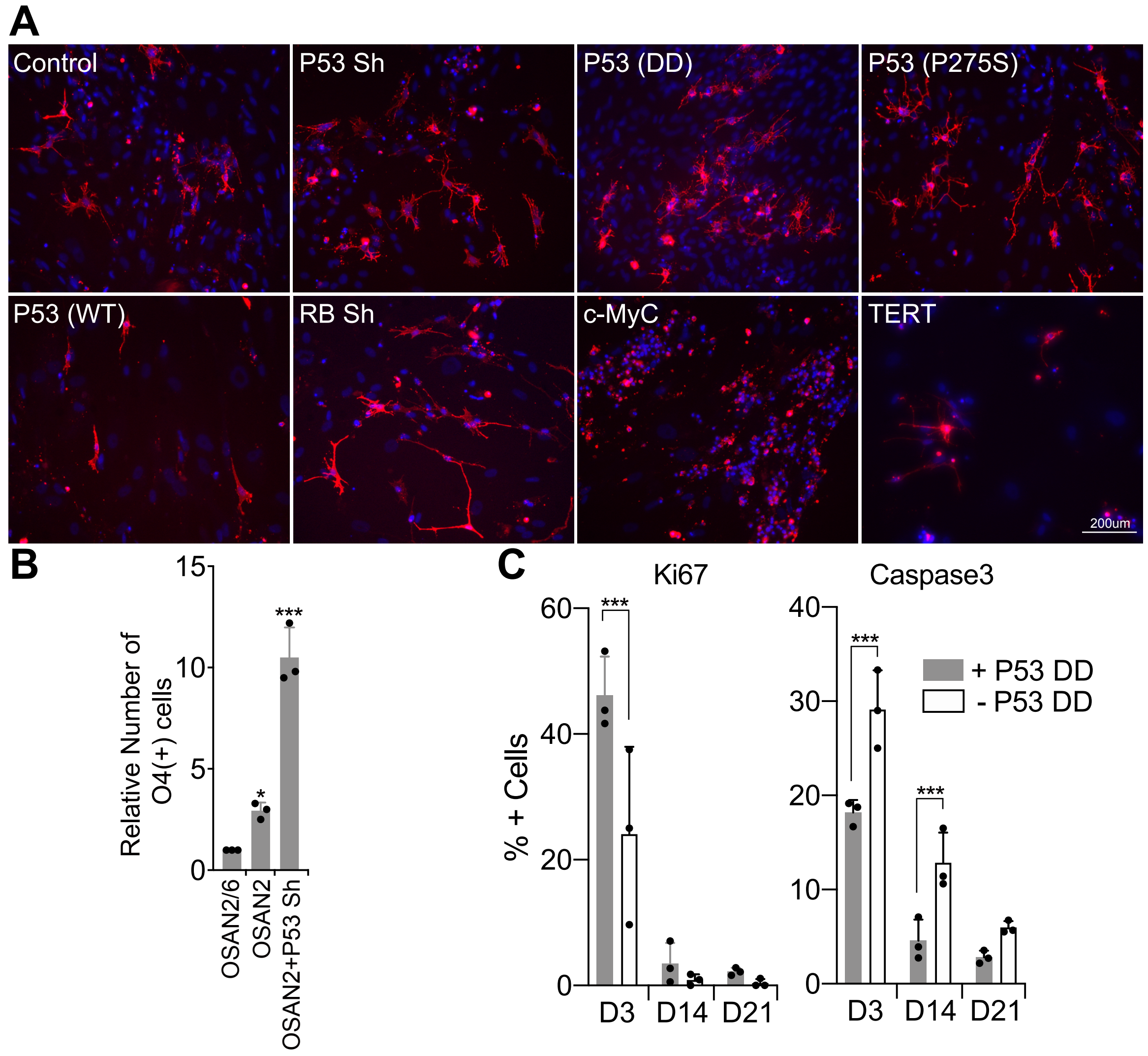

Fig. S4. Improvement of iOPC efficiency by P53 pathway manipulation. (**A**) Representative images of day 21 iOPC culture after P53 inhibition treatments. P53Sh = small hairpin P53, P53DD = carboxy-terminal dominant negative fragment of P53, P53 P275S = dominant negative P53 mutant Pro275Ser, P53 WT = P53 wildtype, RB Sh = small hairpin retinoblastoma protein. (**B**) Quantification of the effect of P53Sh (N = 3, error bars indicate mean ± SD, one way ANOVA, * indicates p-value<0.05, *** indicates p-value<0.001). **(C)** Effect of P53DD on cellular proliferation (Ki67) and cell death (Caspase3). Whereas P53DD increases cellular proliferation in the early phase of reprogramming (Day 3), P53DD continues to decrease cellular death into the mid phase of reprogramming (Day 14).

Development • Supplementary information

Suppl Figure 5

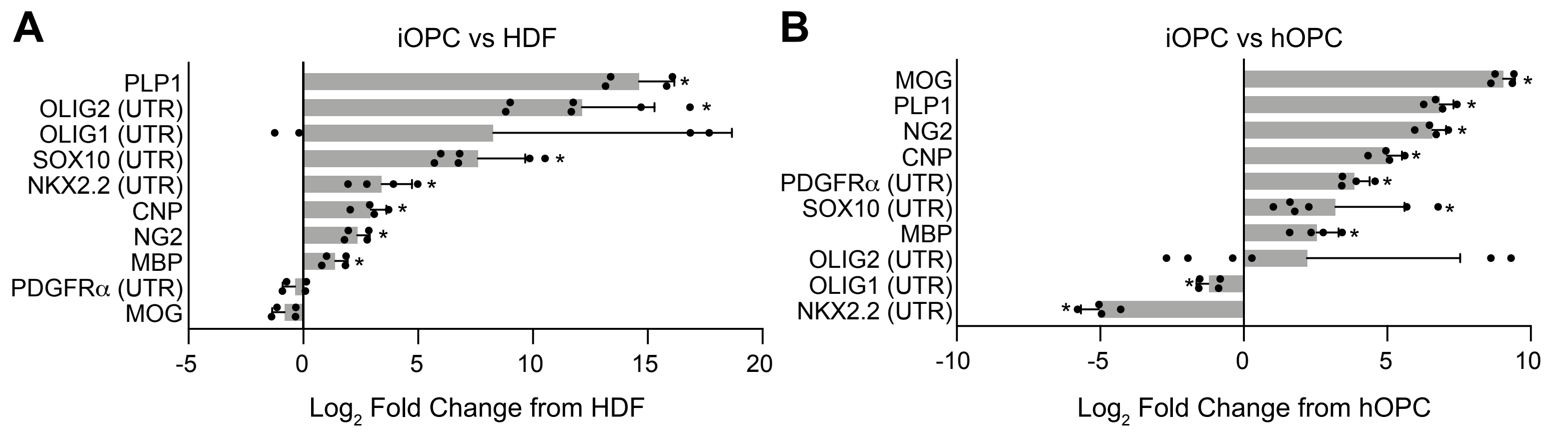

Fig. S5. Quantitative PCR confirmation of RNAseq data. (**A**) Expression levels of selected known oligodendrocyte-specific genes were assessed by qPCR in four-factor iOPC compared to original HDFs, and (**B**) compared to purified human primary OPC. Note untranslated regions (UTR) were used to detect activation of endogenous genes for genes used for reprogramming (N = 4-6, error bars indicate mean± SEM, one sample t-test,* indicates p-value<0.05).

Development • Supplementary information
