# Supplementary material for: Generation of functional human oligodendrocytes from dermal fibroblasts by direct lineage conversion
Source: Development. 2022 Jun 24;149(20):dev199723. doi: 10.1242/dev.199723 (PMC9357374; doi:10.1242/dev.199723)
Supplement: Reviewer comments [file develop-149-199723_review_history.pdf]

Generation of functional human oligodendrocytes from dermal fibroblasts by direct lineage conversion

Koji Tanabe, Hiroko Nobuta, Nan Yang, Cheen Euong Ang, Philip Huie Jr., Sacha Jordan, Michael C. Oldham, David H. Rowitch and Marius Wernig
DOI: 10.1242/dev.199723

Editor: Paola Arlotta

Review timeline

Original submission:	21 April 2021
Editorial decision:	2 January 2022
First revision received:	18 January 2022
Editorial decision:	28 March 2022
Second revision received:	2 May 2022
Accepted:	3 May 2022

Original submission

First decision letter

MS ID#: DEVELOP/2021/199723

MS TITLE: Generation of Functional Human Oligodendrocytes from Dermal Fibroblasts by Direct Lineage Conversion

AUTHORS: Koji Tanabe, Hiroko Nobuta, Nan Yang, Cheen Euong Ang, Philip Huie Jr., Michael C Oldham, David H Rowitch, and Marius Wernig

Dear Marius,

I have now received all the referees' reports on the above manuscript, and have reached a decision. The referees' comments are appended below, or you can access them online: please go to BenchPressand click on the 'Manuscripts with Decisions' queue in the Author Area.

As you will see, the referees express considerable interest in your work, but have some criticisms and recommend a substantial revision of your manuscript before we can consider publication. If you are able to revise the manuscript along the lines suggested, which may involve further experiments, I will be happy receive a revised version of the manuscript. Your revised paper will be re-reviewed by one or more of the original referees, and acceptance of your manuscript will depend on your addressing satisfactorily the reviewers' major concerns. Please also note that Development will normally permit only one round of major revision.

We are aware that you may be experiencing disruption to the normal running of your lab that make experimental revisions challenging. If it would be helpful, we encourage you to contact us to discuss your revision in greater detail. Please send us a point-by-point response indicating where you are able to address concerns raised (either experimentally or by changes to the text) and where you will not be able to do so within the normal timeframe of a revision. We will then provide further guidance. Please also note that we are happy to extend revision timeframes as necessary.

Please attend to all of the reviewers' comments and ensure that you clearly highlight all changes made in the revised manuscript. Please avoid using 'Tracked changes' in Word files as these are lost

© 2022. Published by The Company of Biologists under the terms of the Creative Commons Attribution License (https://creativecommons.org/licenses/by/4.0/).

in PDF conversion. I should be grateful if you would also provide a point-by-point response detailing how you have dealt with the points raised by the reviewers in the 'Response to Reviewers' box. If you do not agree with any of their criticisms or suggestions please explain clearly why this is so.

Many thanks,
Paola Arlotta
Handling Editor
Development

Reviewer 1

Advance summary and potential significance to field

Oligodendrocytes and their progenitors, OPCs, are important cell types in the central nervous system. Cellular models represent essential tools to define OPC/oligodendrocyte development and function and model associated diseases.
However, these cells have been particularly challenging to generate from primary and pluripotent stem cell sources. As such, direct reprogramming approaches provide an avenue to readily access these cells. While mouse iOPCs were first reported by this group and others over 8 years ago, the derivation of human iOPCs has remained challenging. The first clear report of human iOPCs was in April 2021 in Stem Cell Reports. In the current manuscript, the authors validate that human iOPCs can be generated in vitro from human fibroblasts (with slightly different transcription factors) and extend the characterization of human iOPCs to demonstrate in vivo engraftment and myelin generation. The images of iOPC-derived oligodendrocytes are particularly beautiful and nicely show the potential utility of this system.

Comments for the author

Essential revisions:
1. The negative data at the beginning of the manuscript is hard to interpret without more information and data. The first section claims that “Rodent iOPC reprogramming factors do not work for human fibroblasts”. First it isn’t clear if this is to mean the rodent ORF sequences or the human sequences of the same TFs that were previously used in rodent cells. Second, no data is provided to evaluate the claim. Were all the factors expressed and translated appropriately and to what level? Need to add the data or just eliminate this section.
2. The impression that the Moloney-based retroviruses are better could use additional evidence or direct comparison to lentiviral vectors. It isn’t clear if the retro viruses are using the same ORFs as the lentis. It isn’t clear what transcript or protein expression differences between retros and lentis actually made the difference.
3. The computational data of potential OPC factors is out of place in this manuscript and does not add anything in the current form. I suggest removing unless those factors are also tested in the retro vectors.
4. Information on sustained transgene expression levels and dependence is important to understand the reprogramming status of the cells. Is transgene silencing necessary for differentiation to oligodendrocytes?
5. More data or better explanation of the p53 results would be important.
It isn’t clear if these manipulations are simply increasing the proliferation rate of iOPCs or if they play an actual role in the reprogramming process.
6. RNAseq datasets should be available to reviewers. Also, please define the replicates used for RNAseq
7. Additional description and possibly data on the in vivo engraftment would be helpful.
Detailed engraftment studies are likely beyond the scope of this study but understanding the general extent of human cell engraftment and survival would be helpful. It is hard to know if in vivo generation of oligodendrocytes from human iOPCs is an ultra-rare event at this stage of the technology or something that can actually be immediately useful to other labs.
Potential extensions that would enhance manuscript:
8. The claim that it is “impossible” to determine the actual reprogramming efficiency is somewhat overstated. I agree that it is more challenging but cellular barcoding strategies could be

© 2022. Published by The Company of Biologists under the terms of the Creative Commons Attribution License (https://creativecommons.org/licenses/by/4.0/).

employed. It isn't essential for this proof-of-concept manuscript but understanding the actual reprogramming efficiency is/will be important.
9. A barrier to human OPC work is the long differentiation protocols but also challenges with expanding human OPCs and cryopreservation. It would be useful to know if human iOPCs could be expanded or thawed.

Minor comments:
10. Many of the subsections are very short and could be combined for ease of reading.
11. It would be helpful for all bar graphs to show all datapoints so that readers can easily interpret variability.

Reviewer 2

Advance summary and potential significance to field

In this study, Tanabe et al. provide a new protocol for fast and direct induction of oligodendroglial fate in cultured human fibroblasts by overexpressing a cocktail of genes and adding a dominant negative form of the p53 protein. The authors also showed that those induced oligodendrocyte precursor cells (iOPCs) are transcriptionally similar to human OPCs. Finally, authors show that iOPCs are functional and can be transplanted in mice in vivo, where they seem to myelinate surrounding axons, and that could be useful to study disease-related mutations in oligodendrocytes from patients with a hypomyelinating disease.

Comments for the author

In this study, Tanabe et al. provide a new protocol for fast and direct induction of oligodendroglial fate in cultured human fibroblasts by overexpressing a cocktail of genes and adding a dominant negative form of the p53 protein. The authors also showed that those induced oligodendrocyte precursor cells (iOPCs) are transcriptionally similar to human OPCs. Finally, authors show that iOPCs are functional and can be transplanted in mice in vivo, where they seem to myelinate surrounding axons, and that could be useful to study disease-related mutations in oligodendrocytes from patients with a hypomyelinating disease. Overall this new approach seems to be useful for the field, although the efficiency of the conversion is not very high and I have some concerns:
1. The reprogramming efficiency presented in Figure 1 and Suppl Figure 1 is confusing. I guess data in Suppl Figure 1 keeping only the best conditions was also used in Figure 1F. However, while in Figure 1F the reprogramming efficiency in adult fibroblasts is around 2.8%, data in Suppl Figure 1 shows a reprogramming efficiency for this condition is 1.5%. How is this difference explained?
2. It is not clear along the manuscript if authors use adult or neonatal fibroblasts for their experiments.
Given the big difference between both conditions presented in Suppl Figure 1, this should be clarified.
Second, authors mention that there are some culture conditions or gene combinations that fail to induce the conversion. However, some controls with non-infected cultures, or infected with control viruses are missing to ruled out a potential contamination of OPCs or spontaneous conversion of fibroblasts.
3. The data referred to the first section of the results is not shown. Authors should consider adding this data as it could help to increase the relevance of their new protocol.
4. Although their in silico approach is interesting, the fact that it failed to provide better results than the OSAN2/6+ combination makes this point a weak beginning for the paper. I would suggest to check additional published databases that support the use of novel genes (specially Ascl1 and Nkx2.2), such as in Chamling et al., 2021 (Nature Communications). A better justification of the selection of these genes should be included in the paper.
5. The reading of the manuscript is confusing. The description of the results starts with Suppl Figure 2.
Moreover in this figure, OSAN2/6 abbreviature has not been previously explained in the manuscript and is not detailed in the figure legend. I consider that this figure should be placed later on the paper, after all the characterization of OSAN2/6 combination.
6. Regarding Figure 1C, the quantification of the fluorescent intensity does not seem very reliable. Authors should add a control experiment that has not been exposed to any of the

© 2022. Published by The Company of Biologists under the terms of the Creative Commons Attribution License (https://creativecommons.org/licenses/by/4.0/).

retroviral vectors for comparison. In addition, I would suggest performing western blot or RT-qPCR for comparing CD13 expression in control versus infected cultures.

7. In Figure 2C, authors claim that in absence of Nkx2.2, converted cells fail to express PLP1, an oligodendroglial marker. However, there seem to be green signal, corresponding to PLP1 detection in several parts of the images that in addition does not coincide with O4+ cells (oligodendrocytes). How can this be explained?

8. In Figure 2, how was the relative number of O4+ cells calculated in the graphs? Are all the numbers normalized to the OSAN2/6 condition? Why not to a control? Maybe authors could plot the reprogramming efficiency as in Figure 1 to be more consistent.

9. Suppl Fig 4 shows that reprogramming efficiency is influenced by the proportion of each of the virus added. However, the original titer of each virus is not provided. This is important as the size of the construct which mainly depends on the size of the inserted gene, is crucial for the titer of the virus produced.

10. For experiments of cell transplantation, it should be included data of a transplanted area with non-converted fibroblasts as a negative control instead of an area that is not transplanted. In addition, given the relevance of the data, some quantifications could be provided such as the myelin G ratio of axons surrounded by iOPCs and non-converted fibroblasts.

11. Regarding figure 4D-E, it will strengthen the conclusions if authors add some data to show that a defective myelination in PMD patients is also observable in the iOPCs.

Minor points:

1. Figure 1F is cited before than Figure 1D and C. This should be corrected.
2. Suppl Figure 1 could be combined with Figure 1 in order to avoid such a small Figure.
3. Controls in Figure 2 are not explained.
4. Histology section in methods is missing. Also, CD13 antibody reference is not cited.
5. For the RNAseq in Figure 3, how iOPCs were isolated from non-converted fibroblasts and purified is not explained.
6. An “A” in “OSAN2” is missing in Figure 2H.
7. In page 7, line 27, it is cited Fig. 3F, but it should be 3G.
8. Overall, figures and graph formats, including alignment of mean bars and error bars, and the order of the panels in accordance with the text must be revised.

<u>Reviewer 3</u>

Advance summary and potential significance to field

The authors report a strategy for generating oligodendrocyte progenitor cells from human dermal fibroblasts based on retorvirus-mediated mexpression of a cocktail of transcription factors (most of which were previously implicated in oligogliogenesis). These OPCs appear to be similar to primary human OPCs and can myelinate upon graftin in vivo. The authors also employ these OPCs in preliminary disease modeling.

Comments for the author

Main points:

1) What was the rationale for the TF combination for successful OPC generation from dermal fibroblasts? The explanation "most likely" sounds a bit vague, and one wonders why the authors needed a computational approach/microdissection to arrive at this judgement of likelihood.

2) It is extremely unlikely that cells are efficiently transduced by all five retroviruses. Is there any evidence that successfully reprogrammed HDF indeed expressed all five TFs? This is the most important concern about this study from a mechanistic point of view. For example individual or combinations of TF may act in some cells thereby creating a microenvironment for other cells expressing other TF combinations and thereby promote the overall reprogramming success without 5F coexpression. These considerations renders the interpretation of the experiments in which individual TFs were removed from the cocktail, or their relative dose changed (e.g. Sox10) difficult/impossible to interpret. The same is true for OE of MDM2 or P53DD. The cells that express all the listed factors in combination must be extremely rare in these cultures.

3) Does the same factor combination when driven from lentiviruses still fail in inducing efficient conversion into OPC?

© 2022. Published by The Company of Biologists under the terms of the Creative Commons Attribution License (https://creativecommons.org/licenses/by/4.0/).

4) If so, would this provide evidence that reprogramming is more efficient in cycling HDFs? Is it conceivable that retroviruses are better in targeting elite cell types (work from the Zandstra lab [Shakiba N, Fahmy A, Jayakumaran G, McGibbon S, David L, Trcka D, Elbaz J, Puri MC, Nagy A, van der Kooy D, Goyal S, Wrana JL, Zandstra PW. Cell competition during reprogramming gives rise to dominant clones. Science. 2019 Apr 26;364(6438):eaan0925. doi: 10.1126/science.aan0925. Epub 2019 Mar 21. PMID: 30898844.] has suggested that iPS reprogramming is more efficient in neural crest-derived fibroblasts than fibroblasts of other origins)? While addressing this point may be out of scope for this study, it would be important to be raised in the discussion.

Additional minor points
1) The first sentence of the abstract reads awkwardly as oligodendrocytes per se don't serve disease modelling or therapy. The meaning is clear, but at least for a non-native speaker, the formulation is awkward.
2) Is tumorigenic potential still considered a bottleneck in clinical application of ES/iPS-derived cells?
3) I believe the word "unfortunately" associated to a negative finding is meaningless in a scientific context.
4) What was the rationale to extract OPC expression data from mid-gestation neocortex?

First revision

Author response to reviewers' comments

First, we would like to thank all the reviewers for their time and effort spent to critically review our manuscript. We are grateful for the insightful questions, constructive suggestions for improvements, and considerable interests in the manuscript. We have spent a significant amount of time addressing all points. Below, we attached a point-by- point response to all questions and concerns. We hope that you feel they collectively improved the manuscript and will make the paper now acceptable for publication.

Reviewer 1
1.1. The negative data at the beginning of the manuscript is hard to interpret without more information and data. The first section claims that “Rodent iOPC reprogramming factors do not work for human fibroblasts”. First it isn’t clear if this is to mean the rodent ORF sequences or the human sequences of the same TFs that were previously used in rodent cells. Second, no data is provided to evaluate the claim. Were all the factors expressed and translated appropriately and to what level? Need to add the data or just eliminate this section.

Response: We are sorry for the confusion caused by the wording in the manuscript. We clarify here that the same ORF sequences used in rodent fibroblasts were used in the current experiments. It is rather the technical procedure (lentiviral delivery of key TFs) that hindered reprogramming. Please see the response to the next questions for details. To provide supporting evidence for negative result from lentivirus-mediated reprogramming, we have provided an image showing the lack of morphological features of OPCs obtained by this method (Suppl Fig. 1A) and rephrased the paragraph in the manuscript (Pg 3, underlined line now reads “Rodent iOPC reprogramming protocol does not support human fibroblast reprogramming” instead of “Rodent iOPC reprogramming factors do not work for human fibroblasts”).

1.2. The impression that the Moloney-based retroviruses are better could use additional evidence or direct comparison to lentiviral vectors. It isn’t clear if the retro viruses are using the same ORFs as the lentis. It isn’t clear what transcript or protein expression differences between retros and lentis actually made the difference.

Response: Intriguingly, we had made a similar observation (of a superiority of moloney viruses over lenti viruses) early on in iPS cell reprogramming from human fibroblasts (Takahashi, et al., Cell.

© 2022. Published by The Company of Biologists under the terms of the Creative Commons Attribution License (https://creativecommons.org/licenses/by/4.0/).

2007). We believe that reprogramming requires supraphysiological concentrations of reprogramming factors while minimizing cell toxicity caused to stress of viral infection. In new experiments we have directly compared lentivirus and retrovirus infections in HDFs and found that although similar infection efficiency can be achieved, the amount of protein expression is significantly higher from retrovirus infection. Trying to infect with increased amounts of lentivirus to achieve comparable transgene expression levels caused substantial cell death. This may highlight and explain the reason why a lentivirus-mediated reprogramming was not successful. This information has been added to Suppl Fig. 1B-D and text Pg. 4 (underlined).

1.3. The computational data of potential OPC factors is out of place in this manuscript and does not add anything in the current form. I suggest removing unless those factors are also tested in the retro vectors.
Response: We clarify here that yes, we did test these factors in retrovirus vectors. We learned that although the candidate genes resulted from computational analysis were highly specific to OPCs, they were not highly expressed. Overexpressing such genes by retrovirus resulted in negative findings. We are happy to remove the data if the reviewers insist, but given the identification of a new set of OL-specific genes, we would prefer to keep the data in the manuscript in order to publish and make available this interesting set of genes to the scientific community.

1.4. Information on sustained transgene expression levels and dependence is important to understand the reprogramming status of the cells. Is transgene silencing necessary for differentiation to oligodendrocytes?

Response: This is an excellent mechanistic question to the iOPC reprogramming. We observe that the transgenes are not silenced at the end of reprogramming in our protocol as shown here. So silencing is not a requirement for successful reprogramming.

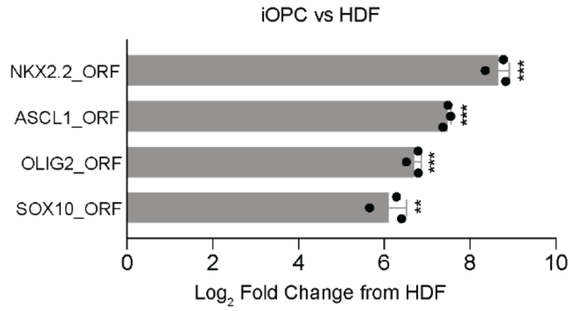

1.5. More data or better explanation of the p53 results would be important. It isn't clear if these manipulations are simply increasing the proliferation rate of iOPCs or if they play an actual role in the reprogramming process.

Response: We are thankful for this question and now provide in a new figure the quantification for cellular proliferation during iOPC conversion with or without P53DD, using Ki67 as a proliferation marker (Suppl Fig. 3C). In this figure, we systematically tested the effect of P53DD on proliferation as well as cell death (Caspase-3 as a marker) throughout the reprogramming time course. Our observations suggest that during the initial 3 days of reprogramming, P53DD increased the proliferation rate of HDFs but the effect diminished at mid- (day 14) and late- (day 21) phases of reprogramming. Because we do not observe iOPCs until after day 14, P53DD does not seem to increase the proliferation rate of iOPCs themselves, to directly answer your question. In addition, even in presence of P53DD, the proliferation rate diminishes in culture as reprogramming proceeds, further providing evidence that the simple increase in iOPC proliferation is not the cause for increased reprogramming efficiency. On the other hand, the cell death rate is significantly decreased by P53DD in early-to-mid phases of reprogramming. Therefore, we suspect that the beneficial effect of P53DD in reprogramming efficiency is more related to reduced cell death than increased proliferation. This point has been included in the discussion section (Pg. 6, underlined).

1.6. RNAseq datasets should be available to reviewers. Also, please define the replicates used for RNAseq

© 2022. Published by The Company of Biologists under the terms of the Creative Commons Attribution License (https://creativecommons.org/licenses/by/4.0/).

Response: We are happy to provide the RNAseq datasets to the reviewers. Please access them at: https://docs.google.com/spreadsheets/d/1rBNCAzioYVmJmfxqXvDJXVOpVjtYFWMC/edit?usp=sharing&ouid=104168382845403685280&rtpof=true&sd=true.

We clarify that each group (iOPC, human forebrain purified primary OPC, human iPS cell- derived OPCs) needed to be isolated from a bulk culture/tissues and pooled in order to obtain sufficient number of cells. Therefore the data presented were shown as single sample size per group, but each was obtained from pooled, multiple experiments.

1.7. Additional description and possibly data on the in vivo engraftment would be helpful. Detailed engraftment studies are likely beyond the scope of this study but understanding the general extent of human cell engraftment and survival would be helpful. It is hard to know if in vivo generation of oligodendrocytes from human iOPCs is an ultra-rare event at this stage of the technology or something that can actually be immediately useful to other labs.

Response: This is an excellent point. The engrafted cells are scarce but consistently found in transplanted animals. Unfortunately, we have not determined how many cells survive the transplantation procedure and we have not saved serial sections therefore cannot reconstruct the entire engraftment sites. Trying to estimate, we see up to a dozen or so MBP+ cells in a relatively large area within a 20µm slice, so we roughly estimate the total number of properly matured and engrafted cells per mouse to be in the low hundreds. Assuming that on average 10% of the d21 cells are O4+ iOPCs and the immediate transplantation survival is 20%, the fraction of properly long term (12 weeks) incorporated oligodendrocytes is around 10%. We would like to note that this low estimated engraftment efficiency should be seen in the light that the iOPCs on day 21 do not seem to be highly proliferative and presumably become postmitotic right after transplantation.

Potential extensions that would enhance manuscript:
1.8. The claim that it is “impossible” to determine the actual reprogramming efficiency is somewhat overstated. I agree that it is more challenging but cellular barcoding strategies could be employed. t isn’t essential for this proof-of-concept manuscript but understanding the actual reprogramming efficiency is/will be important.

Response: Thank you for suggesting a great experiment; it is in fact a feasible experiment, but we feel the efforts required would exceed the benefit. For the current manuscript, we provided one quantification method (Fig. 1C, Suppl Fig. 1F) based on the number of initially seeded cells. We have changed the wording of ‘impossible’ in the text (Pg 4, underlined).

1.9. A barrier to human OPC work is the long differentiation protocols but also challenges with expanding human OPCs and cryopreservation. It would be useful to know if human iOPCs could be expanded or thawed.

Response: We are grateful for this highly relevant topic. We provide the proliferative capacity of iOPC in a new figure Supple Fig 3C using Ki67 as a proliferation marker. During the initial phase of reprogramming, cells are confirmed to be proliferating, but just like primary OPCs, they are sensitive to freeze/thaw stress and in our hands, we clarify that iOPC cannot be frozen/thawed for future use.

Minor comments:
1.10. Many of the subsections are very short and could be combined for ease of reading.

Response: Thank you for the comment - we have combined a couple of subsections (Pg 4-5) for the ease of reading.

1.11. It would be helpful for all bar graphs to show all datapoints so that readers can easily interpret variability.

Response: Thank you for the suggestion. We have changed all graphs to dotplots.

© 2022. Published by The Company of Biologists under the terms of the Creative Commons Attribution License (https://creativecommons.org/licenses/by/4.0/).

Reviewer 2 Comments for the Author:
In this study, Tanabe et al. provide a new protocol for fast and direct induction of oligodendroglial fate in cultured human fibroblasts by overexpressing a cocktail of genes and adding a dominant negative form of the p53 protein. The authors also showed that those induced oligodendrocyte precursor cells (iOPCs) are transcriptionally similar to human OPCs. Finally, authors show that iOPCs are functional and can betransplanted in mice in vivo, where they seem to myelinate surrounding axons, and that could be useful to study disease-related mutations in oligodendrocytes from patients with a hypomyelinating disease. Overall, this new approach seems to be useful for the field, although the efficiency of the conversion is not very high, and I have some concerns:

2.1. The reprogramming efficiency presented in Figure 1 and Suppl Figure 1 is confusing. I guess data in Suppl Figure 1 keeping only the best conditions was also used in Figure 1F. However, while in Figure 1F the reprogramming efficiency in adult fibroblasts is around 2.8%, data in Suppl Figure 1 shows a reprogramming efficiency for this condition is 1.5%. How is this difference explained?
Response: We clarify here that the data shown in Fig. 1C and Suppl Fig. 1F were obtained from independent experiments. We therefore did not omit or show partial data used in Fig. 1. Although the same fibroblast lines were used and experiments were done in triplicates, on average, we do see some variability depending on the batch of experiment, potentially a higher passage number of the (primary) fibroblast line may influence the reprogramming efficiency as well.

2.2 It is not clear along the manuscript if authors use adult or neonatal fibroblasts for their experiments. Given the big difference between both conditions presented in Suppl Figure 1, this should be clarified. Second, authors mention that there are some culture conditions or gene combinations that fail to induce the conversion. However, some controls with non-infected cultures, or infected with control viruses are missing to ruled out a potential contamination of OPCs or spontaneous conversion of fibroblasts.

Response: We thank the reviewer to bring up this important point. We first clarify that except the experiment that compared adult vs neonatal fibroblasts in reprogramming efficiency (Suppl Fig. 1F), we used neonatal fibroblasts of various sources throughout the manuscript. We agree that we should not exclude the possibility of spontaneous conversion without reprogramming factors, due maybe to the components in the favorable OPC medium. We therefore added a control group infected with a GFP virus and confirmed that the reprogramming does not occur in this condition (Fig. 1C, Suppl Fig. 1E).

2.3 The data referred to the first section of the results is not shown. Authors should consider adding this data as it could help to increase the relevance of their new protocol.

Response:
We assume this question is referring to our initial observation that lentiviruses do not reprogram HDFs to iOPCs. We agree with the reviewer and have added supporting evidence that HDFs infected with the lentiviruses encoding the reprogramming factors do not show morphological features of OPC (Suppl Fig. 1A). In addition, to better understand the advantage of retrovirus- mediated reprogramming, we made a direct comparison between lentivirus and retrovirus infections in HDFs and found that although similar infection efficiency can be achieved, the amount of protein expression is significantly higher from retrovirus infection. Trying to infect with increased amounts of lentivirus caused significant amount of cell death. This may highlight and explain the reason why a lentivirus-mediated reprogramming was not successful. This information has been added to Suppl Fig. 1B-D, and text Pg. 4 (underlined).

2.4 Although their in silico approach is interesting, the fact that it failed to provide better results than the OSAN2/6+ combination makes this point a weak beginning for the paper. I would suggest to check additional published databases that supports the use of novel genes (specially Ascl1 and Nkx2.2), such as in Chamling et al., 2021 (Nature Communications). A better justification of the selection of these genes should be included in the paper.

Response: We agree with the reviewer that the in silico approach that we incorporated during TF selection led to a negative results. However, if the reviewer is OK with that, we would like to keep these data in the manuscript for two reasons: First, even negative results may be worth reporting as other scientists in the field may be interested in the outcome of some of these OL- specific

© 2022. Published by The Company of Biologists under the terms of the Creative Commons Attribution License (https://creativecommons.org/licenses/by/4.0/).

genes and second, many of the in silico predicted genes appear to be novel OL-specific genes and the community might be interested in this information and potentially follow up on their function in oligodendrocyte biology. In regards to the selection of ASCL1 and NKX2.2, they are indeed well-known, important genes for oligodendrocyte specification and differentiation especially in the spinal cord (Sugimori et al., Development. 2008; Fu et al., Development. 2002; Vue et al., Development. 2014; Parras et al., J Neurosci. 2007; Danesin et al., Development. 2001). In addition, ASCL1 and NKX2.2 are highly expressed genes in OPC population along with our other selected genes according to the RNAseq data comparing OPC, immature, mature oligodendrocytes, neurons, and astrocytes (Zhang et al., J Neurosci 2014).

2.5 The reading of the manuscript is confusing. The description of the results starts with Suppl Figure 2. Moreover in this figure, OSAN2/6 abbreviature has not been previously explained in the manuscript and is not detailed in the figure legend. I consider that this figure should be placed later on the paper, after all the characterization of OSAN2/6 combination.
Response: We apologize for the confusion. We have rearranged the figure order so that the manuscript starts with Suppl Fig. 1 and clarified the abbreviation in the text & figure legend.

2.6 Regarding Figure 1C, the quantification of the fluorescent intensity does not seem very reliable. Authors should add a control experiment that has not been exposed to any of the retroviral vectors for comparison. In addition, I would suggest performing western blot or RT- qPCR for comparing CD13 expression in control versus infected cultures.

Response: We thank the reviewer for pointing to the missing control group. We have added quantification of CD13 on D0 (before reprogramming) as a baseline control. The quantification of the fluorescent intensity was obtained by capturing images in a CCD camera with all settings being equal across time course, opening the raw images without manipulating brightness/contrast/levels in Fiji (formally ImageJ), then measuring mean fluorescent intensity in the built-in analysis. We therefore think we took steps to minimize error in this quantification.
Regardless, we have performed the proposed experiments and now provide additional data that in addition to CD13 protein, other fibroblast signature genes (*ANPEP [CD13], MMP1, DCN, LUM, CLDN11, FN1, CAV1, COL6A2, S100A6, CTSK, VIM*) are also downregulated after iOPC induction at the mRNA level (Fig. 1F). These additional data on protein and mRNA level, we would say, further strengthen the notion that fibroblast identity is decreased during iOPC induction.

2.7 In Figure 2C, authors claim that in absence of Nkx2.2, converted cells fail to express PLP1, an oligodendroglial marker. However, there seem to be green signal, corresponding to PLP1 detection in several parts of the images that in addition does not coincide with O4+ cells (oligodendrocytes). How can this be explained?

Response: We thank the reviewer for pointing to the existence of faint PLP1 signal. We clarify here that there is a faint, background level of PLP1 staining in the fibroblast. It is apparent in all conditions (panels) in Fig 2C and is much lower level compared to the O4+ iOPCs. We clarify that the PLP1 gene expression is negligible measured by qPCR and RNA sequencing, therefore we believe this background signal is due to a low level nonspecific binding of the antibody. We do note that the difference between the background level and the signal that colocalizes with O4 is large and obviously detected.

2.8 In Figure 2, how was the relative number of O4+ cells calculated in the graphs? Are all the numbers normalized to the OSAN2/6 condition? Why not to a control? Maybe authors could plot the reprogramming efficiency as in Figure 1 to be more consistent.

Response: Thank you for pointing to this confusion. We clarify that all numbers were compared to the OSAN2/6 condition. We normalized to that because we wanted to emphasize the improvements from the baseline condition. To make this point clearer, we now show fold change over the OSAN2/6 condition in the revised figure panels (Fig. 2B, D-F).

2.9 Suppl Fig 4 shows that reprogramming efficiency is influenced by the proportion of each of the virus added. However, the original titer of each virus is not provided. This is important as the size of the construct, which mainly depends on the size of the inserted gene, is crucial for the titer of the virus produced.

© 2022. Published by The Company of Biologists under the terms of the Creative Commons Attribution License (https://creativecommons.org/licenses/by/4.0/).

Response: The reviewer is absolutely correct in this point. We did not measure the titer of viruses during our experiment due to the fact that titers are greatly affected by the cell types and culture conditions. In our experience a MOI in 293T cells has little predictive value for fibroblasts. It is therefore better to titer the virus in the experimental cell type. We have hence quantified the actual infection efficiencies in human fibroblasts for each virus. We obtained the following results: OLIG2 = 76.47%, SOX10 = 90.65%, ASCL1 = 100%, NKX2.2 = 86.87%, as determined by immunohistochemistry. Thus, the unique effect of doubling the amount of SOX10 viruses does not appear to simply because of a low Sox10 virus titer but rather suggests higher protein levels per cell are needed. This information was added to the Methods (underlined).

2.10 For experiments of cell transplantation, it should be included data of a transplanted area with non-converted fibroblasts as a negative control instead of an area that is not transplanted. In addition, given the relevance of the data, some quantifications could be provided such as the myelin G ratio of axons surrounded by iOPCs and non-converted fibroblasts.

Response: We and others have transplanted unperturbed fibroblasts into mouse brains and never observed convincing evidence of transdifferentiation into neural cell types including myelinating oligodendrocyte formation (Steffel et al., Stem Cells 2003, Zhang et al., J Neurotrauma. 2005, Pin-Chun, et al., Brain Behav. 2019, Tuszynski et al., Exp Neurol. 1994).
To the best of our knowledge, transdifferentiation of fibroblasts into authentic neural cell types does not occur spontaneously and requires the expression of exogenous reprogramming factors. In an extreme example, cell fusion with neurons has been found to be the explanation for the original claim of neuronal transdifferentiation from hematopoietic cells without exogenous expression of reprogramming factors (Brazelton et al., Science 2000). Simple fusion of transplanted human fibroblasts with endogenous oligodendrocytes without transdifferentiation can be excluded in our case, because we detect MBP immunereactivity in transplanted cells which can only be derived from human myelinating oligodendrocytes that produce human MBP since this gene is absent in all cells in the recipient *Shiverer* mice. Based on these considerations, we feel there will be little information gained from such additional animal experiments. With regards to quantification of myelination by iOPCs, we followed the reviewer's advice and quantified G-ratios from16 myelinated axons found in the areas of transplanted iOPCs and found a significant increase in the average G-ratio compared to endogenous axons in the un-transplanted areas. The text has been updated to include this information (Pg. 8, underlined)

2. 11.Regarding figure 4D-E, it will strengthen the conclusions if authors add some data to show that a defective myelination in PMD patients is also observable in the iOPCs.

Response: We agree that assessment of myelination for the disease modeling would further strengthen the paper. However, to best address this question it would require quite an extensive transplantation experiment that we feel is beyond the scope of this study. The pronounced decrease in oligodendrocyte viability observed further complicates the interpretation of transplantation data as seen in PMD patient-derived iPS cell-derived OPCs (Nobuto et al., 2019 Cell Stem Cell). For these reasons we politely request not to perform such involved animal experiments.

Minor points:

2.12. Figure 1F is cited before than Figure 1D and C. This should be corrected.

Response: Thank you for pointing this out. We have corrected in the text.

2.13. Suppl Figure 1 could be combined with Figure 1 in order to avoid such a small Figure.

Response: Thank you for the suggestion. In response to reviewer comments, Suppl Fig1 now has more panels, therefore we kept the original Suppl Fig1 data as is.

2.14. Controls in Figure 2 are not explained.

Response: Thank you for pointing to the need for clarification. We have now re-labeled the control as “OSAN2/6” and added clarification in the text (Pg. 5, underlined).

© 2022. Published by The Company of Biologists under the terms of the Creative Commons Attribution License (https://creativecommons.org/licenses/by/4.0/).

2.15. Histology section in methods is missing. Also, CD13 antibody reference is not cited.

Response: Thank you for pointing this out. We have added the “Histology & image acquisition” section and antibody information (BD Biosciences 557454) in the methods.

2.16. For the RNAseq in Figure 3, how iOPCs were isolated from non-converted fibroblasts and purified is not explained.

Response: Thank you for pointing this out. We have added “iOPC isolation” section in the methods.
2.17. An “A” in “OSAN2” is missing in Figure 2H.

Response: Thank you for pointing this out. We have corrected in the figure.

2.18.In page 7, line 27, it is cited Fig. 3F, but it should be 3G.

Response: Thank you for pointing this out. We have corrected in the typo.
2.19.Overall, figures and graph formats, including alignment of mean bars and error bars, and the order of the panels in accordance with the text must be revised.

Response: We have adjusted the order of panels and alignments in graphs.

Reviewer 3 Comments for the Author:
Main points:
3.1) What was the rationale for the TF combination for successful OPC generation from dermal fibroblasts? The explanation "most likely" sounds a bit vague, and one wonders why the authors needed a computational approach/microdissection to arrive at this judgement of likelihood.

Response: The final TF combination was accomplished through systematic interrogation of TFs known to be important for oligodendrocyte biology as well as a list of candidates nominated by a computational approach developed by Mike Oldham and TFs previously tried in mouse fibroblast>iOPC reprogramming. We used expression of O4+ cells as main initial criteria to assess reprogramming capability as this marker needs to be induced in primary OPCs. We then applied morphological criteria, as oligodendrocytes have a very characteristic morphology which in combination with O4-positivity presumably integrates several cell biological characteristics of oligodendroglial identity. Our morphological criteria were trained by data from human primary OPCs before and after differentiation. We agree the wording ‘most likely’ was non-ideal, we have re-phrased the sentence which reads now, “*We settled down to the five transcription factors OLIG2, SOX10, ASCL1, NKX2.2, and NKX6.1 (referred as OSAN2/6) based on appearance of O4+ cells as well as morphological characteristics resembling human primary OPCs during various combinatorial TF applications* “ (Pg. 4, underlined).

3.2) It is extremely unlikely that cells are efficiently transduced by all five retroviruses. Is there any evidence that successfully reprogrammed HDF indeed expressed all five TFs? This is the most important concern about this study from a mechanistic point of view. For example, individual or combinations of TF may act in some cells thereby creating a microenvironment for other cells expressing other TF combinations and thereby promote the overall reprogramming success without 5F coexpression. These considerations renders the interpretation of the experiments in which individual TFs were removed from the cocktail, or their relative dose changed (e.g. Sox10) difficult/impossible to interpret. The same is true for OE of MDM2 or P53DD. The cells that express all the listed factors in combination must be extremely rare in these cultures.

Response: Thank you for raising this important question regarding iOPC reprogramming mechanism. We now provide quantification of infection rates of all transcription factors to calculate the fraction of cells expressing all 4 factors. We observed the following infection efficiencies measured by immunohistochemistory: OLIG2 = 76.47%, SOX10 = 90.65%, ASCL1 = 100%, NKX2.2 = 86.87%. This calculates that about 76.47% x 90.65% x 100% x 86.87% = 60.22% cells are infected with all 4 factors. The true infection rates are likely higher because our immunostain assay will have a limit of detection. Thus, it is in fact the majority of the cells which is infected by all viruses. This information was added to the Methods.

© 2022. Published by The Company of Biologists under the terms of the Creative Commons Attribution License (https://creativecommons.org/licenses/by/4.0/).

It is an intriguing concept raised by the reviewer that microenvironment of the surrounding cells infected by other TFs promote reprogramming of other cells. It is also known that environmental factors can influence other reprogramming systems. However, from what is currently known about TFs during reprogramming the most important reprogramming mechanisms are pioneer factor activity and/or co-operative binding at target loci, both of which requires cell-autonomous action on the chromatin.

3.3) Does the same factor combination when driven from lentiviruses still fail in inducing efficient conversion into OPC?

Response: The reviewer is correct. The same factor combination deliver by lentiviruses fails to convert human fibroblasts into iOPCs efficiently. Perhaps related, also iPS cell reprogramming efficiency by Moloney-based retroviral delivery also dramatically exceeds lentiviral delivery (Takahashi, et al., Cell. 2007). Our working model is that reprogramming requires supraphysiological concentrations of reprogramming factors and the balance between high cellular expression levels and cell survival is more favorable with Monloney-based retroviruses than lentiviruses. To provide supporting evidence, we made a direct comparison between lentivirus and retrovirus infections in HDFs and found that although similar infection efficiency can be achieved, the amount of protein expression is significantly higher after retrovirus infection. Increasing the titer of lentiviruses caused significant amount of cell death. Thus, the toxicity in our lentiviral preparation prevents to achieve the protein expression levels seen by Moloney-retrovirus preparations. This may highlight and explain the reason why a lentivirus- mediated reprogramming was not successful. This information has been added to Suppl Fig. 1B-D, and text Pg. 4 (underlined).

3.4) If so, would this provide evidence that reprogramming is more efficient in cycling HDFs? Is it conceivable that retroviruses are better in targeting elite cell types (work from the Zandstra lab [Shakiba N, Fahmy A, Jayakumaran G, McGibbon S, David L, Trcka D, Elbaz J, Puri MC, Nagy A, van der Kooy D, Goyal S, Wrana JL, Zandstra PW. Cell competition during reprogramming gives rise to dominant clones. Science. 2019 Apr 26;364(6438):eaan0925. doi: 10.1126/science.aan0925. Epub 2019 Mar 21. PMID: 30898844.] has suggested that iPS reprogramming is more efficient in neural crest-derived fibroblasts than fibroblasts of other origins)? While addressing this point may be out of scope for this study, it would be important to be raised in the discussion.

Response: We agree with the reviewer regarding the advantage of cycling cells for reprogramming. Perhaps the reason why human fibroblast lines from neonatal donors have higher reprogramming efficiencies than ones from adult donors (Fig. 1C) is related to the cycling rate of the cells. Another potential reason is the fact that human fibroblasts in general have higher infection efficiency to retroviruses than lentiviruses, although the mechanism is unknown. We included these points into our revised manuscript on pages 4 and 5 (underlined).

Additional minor points

3.5) The first sentence of the abstract reads awkwardly as oligodendrocytes per se don't serve disease modelling or therapy. The meaning is clear, but at least for a non-native speaker, the formulation is awkward.

Response: We have updated the sentence to “Oligodendrocytes, the myelinating cells of the central nervous system, possess great potential for disease modeling and cell transplantation-based therapies of genetic and acquired leukodystrophies.”

3.6) Is tumorigenic potential still considered a bottleneck in clinical application of ES/iPS-derived cells?

Response: In our interactions with the FDA (in another iPS cell project) the agency is clearly very concerned about the tumorigenic potential of contaminating iPS cells and requested from us extensive long term animal studies to demonstrate that the iPS cell-derived cell population meant to be grafted has no tumorigenic potential even when we could demonstrate an exceedingly low detection limit of our iPS cell detection assay.

© 2022. Published by The Company of Biologists under the terms of the Creative Commons Attribution License (https://creativecommons.org/licenses/by/4.0/).

3.7) I believe the word "unfortunately" associated to a negative finding is meaningless in a scientific context.

Response: We have updated the wording (Pg. 3 & 9)

3.8) What was the rationale to extract OPC expression data from mid-gestation neocortex?
Response: Human oligodendrocytes first appear in mid-gestation (around gestational week 17). We intended to compare iOPCs to the human OPCs during development which have known capacity to myelinate axons, rather than adult-derived OPCs with low remyelinating capacity.
We therefore figured that midgestation is most appropriate and the best standard for comparison.

Second decision letter

MS ID#: DEVELOP/2021/199723

MS TITLE: Generation of Functional Human Oligodendrocytes from Dermal Fibroblasts by Direct Lineage Conversion

AUTHORS: Koji Tanabe, Hiroko Nobuta, Nan Yang, Cheen Euong Ang, Philip Huie Jr., Sacha Jordan, Michael C Oldham, David H Rowitch, and Marius Wernig

I am very sorry that it took longer than expected for me to receive the comments of the reviewers. I have tried to chase them down and today I have two of them. You can see that one of the reviewers is suggesting some final minor modifications, which I hope you can attend to. Once you perform these minor revisions I will personally look this over, without furhh the et review. Thank you for your help finalizing. The manuscript for acceptance.

The referees' comments are appended below, or you can access them online: please go to BenchPress and click on the 'Manuscripts with Decisions' queue in the Author Area.

The overall evaluation is positive and we would like to publish a revised manuscript in Development, provided that the referees' comments can be satisfactorily addressed. Please attend to all of the reviewers' comments in your revised manuscript and detail them in your point-by-point response. If you do not agree with any of their criticisms or suggestions explain clearly why this is so. If it would be helpful, you are welcome to contact us to discuss your revision in greater detail. Please send us a point-by-point response indicating your plans for addressing the referee's comments, and we will look over this and provide further guidance.

Reviewer 2

Advance summary and potential significance to field

In this study, Tanabe et al. provide a new protocol for fast and direct induction of oligodendroglial fate in cultured human fibroblasts by overexpressing a cocktail of genes and adding a dominant negative form of the p53 protein. The authors also showed that those induced oligodendrocyte precursor cells (iOPCs) are transcriptionally similar to human OPCs. Finally, authors show that iOPCs are functional and can betransplanted in mice in vivo, where they seem to myelinate surrounding axons, and that could be useful to study disease-related mutations in oligodendrocytes from patients with a hypomyelinating disease.

Comments for the author

In this study, Tanabe et al. provide a new protocol for fast and direct induction of oligodendroglial fate in cultured human fibroblasts by overexpressing a cocktail of genes and adding a dominant negative form of the p53 protein. The authors also showed that those induced oligodendrocyte precursor cells (iOPCs) are transcriptionally similar to human OPCs. Finally, authors show that

© 2022. Published by The Company of Biologists under the terms of the Creative Commons Attribution License (https://creativecommons.org/licenses/by/4.0/).

iOPCs are functional and can betransplanted in mice in vivo, where they seem to myelinate surrounding axons, and that could be useful to study disease-related mutations in oligodendrocytes from patients with a hypomyelinating disease. Overall, this new approach seems to be useful for the field and the revised version addressed many of the points I addressed in the first review, however, I still think that there are a few points that still need attention:

Point 1. In this revised version, the difference between figure 1C and suppl Figure 1F is not present anymore.
Still, if the variability between the old and the new version of suppl figure 1F is justifiable as the authors claim it might be a problem for the credibility of the rest of the data presented. In fact, it is logical to think that also other values might be affected by this variability hindering their significance.
Point 2. Thank you for the clarification but I still believe that it should be specified in the manuscript.
Point 4. Even though I agree on the importance of publishing negative results I still believe that they shouldn't be positioned in the first paragraphs. Even if the point is to follow your rationale. Also, it is still very unclear how, after all the introductory work on the identification of good candidate TFs for the reprogramming the authors ended up identifying the osan2/6 combination just by "various combinatorial TF application". If it's true that nkx2.2 and ascl1 are known TFs for oligodendrocytes reprogramming, it should be specified in the manuscript.
Point 8. What happened with the graphs containing the added controls?
Point 10. Thank you for addressing my doubts on the negative control. Still, I believe it would be better to include a graph relative to the G-ratio quantification in figure 4.
Point 11. I understand the motivation brought by the authors, but I believe it weakens the impact of this work.
All the minor points have been addressed.
Additional comments:
- The bioinformatic analysis relative to the co-expression association is unclear and confusing. The whole paragraph should be reformulated.
- Why was the p53DD construct chosen even if figures 2D and 2F show higher efficiency for the p53sh construct? Moreover, figures 2D/2F are a confusing as the OSAN2/6 combination seems more effective than the OSAN2+ one, contrary to the author's previous statement.
- Is the SOX10 finding worth a dedicated paragraph?
- How is it explained the high variability in the reprogramming efficiency in figure 2H?

Reviewer 3

Advance summary and potential significance to field

The authors report a strategy for generating induced oligodendrocyte progenitor cells from human dermal fibroblasts based on retrovirus-mediated expression of OLIG2, SOX10, ASCL! and NKX2.2. These OPCs resemble primary human OPCs and can myelinate upon grafting in vivo. The authors also employ these OPCs in modeling a hypomyelinating leukodystrophy.

Comments for the author

All my concerns were addressed.

© 2022. Published by The Company of Biologists under the terms of the Creative Commons Attribution License (https://creativecommons.org/licenses/by/4.0/).

Third decision letter

MS ID#: DEVELOP/2021/199723

MS TITLE: Generation of Functional Human Oligodendrocytes from Dermal Fibroblasts by Direct Lineage Conversion

AUTHORS: Koji Tanabe, Hiroko Nobuta, Nan Yang, Cheen Euong Ang, Philip Huie Jr., Sacha Jordan, Michael C Oldham, David H Rowitch, and Marius Wernig
ARTICLE TYPE: Research Article

I am happy to tell you that your manuscript has been accepted for publication in Development, pending our standard ethics checks.

© 2022. Published by The Company of Biologists under the terms of the Creative Commons Attribution License (https://creativecommons.org/licenses/by/4.0/).
